# Sex Differences in Case Fatality Rate of Patients With Severe Fever With Thrombocytopenia Syndrome

**DOI:** 10.3389/fmicb.2021.738808

**Published:** 2021-10-14

**Authors:** Jing Zhao, Qing-Bin Lu, Hao Li, Yang Yuan, Ning Cui, Chun Yuan, Xiao-Ai Zhang, Zhen-Dong Yang, Shi-Man Ruan, Lan-Zheng Liu, Juan Du, Li-Qun Fang, Wei Liu

**Affiliations:** ^1^State Key Laboratory of Pathogen and Biosecurity, Beijing Institute of Microbiology and Epidemiology, Beijing, China; ^2^Department of Laboratorial Science and Technology, School of Public Health, Peking University, Beijing, China; ^3^The 990th Hospital of Joint Logistic Support Force of Chinese People’s Liberation Army, Xinyang, China; ^4^Jinan Center for Disease Control and Prevention, Jinan, China; ^5^Beijing Key Laboratory of Vector Borne and Natural Focus Infectious Diseases, Beijing, China

**Keywords:** SFTS, sex, age, comorbidity, immune response, fatal outcome

## Abstract

**Background:** Severe fever with thrombocytopenia syndrome (SFTS) is a tick-borne disease with high mortality. However, detailed analysis is lacking to explore the complex effect of sex with age or comorbidities.

**Methods:** A retrospective cohort study was performed among 2,938 SFTS patients entered during 2011–2020 in Xinyang, China. The case fatality rate (CFR) was estimated for their association with sex, age, and comorbidities by an interactive way. The difference of immune response between sex was explored in an age dependent way.

**Results:** An overall CFR of 15.3% (450/2,938) was obtained, which appeared to be higher in males than in females [17.7% vs. 13.6%, adjusted odds ratio (aOR) = 1.24; 95% CI, 1.00–1.53; *P* = 0.048] and increased dramatically with age (*P* < 0.001). The associations between sex and SFTS fatal outcome were age-dependent and varied according to the status of comorbidities. The mortality-related risk conferred by older age was more pronounced in males, with aOR (95% CI) to be 5.76 (3.75–8.84) vs. 5.30 (3.54–7.95) in female. Sex-stratified analysis disclosed significant associations between death and comorbidities among female patients (aOR = 1.87, 95% CI: 1.40–2.49; *P <* 0.001), while none among males. Among females, the significant associations between presence of comorbidity and fatal outcome differed among age groups, with aOR (95% CI) decreased from 2.28 (1.16–4.46) in ≤60 years, to 2.06 (1.34–3.18) in 60–70 years and further to 1.55 (0.97–2.47) in >70 years. Altogether 194 SFTS patients were randomly selected for the test of B cells, natural killer (NK) cells, CD4 cells percentages, and anti-SFTSV IgM antibody level, the results revealed that males >60 years had significantly decreased percentages of B cells, CD4 cells, lower anti-SFTSV IgM antibody titer, and increased level of NK cells than male aged ≤60 years, while none of these age specific differences was observed in the females. This finding underlies the more pronounced age specific difference in CFR among male than female.

**Conclusions:** Males had a significantly higher mortality of SFTS than did females, and more likely to be affected by aging for SFTS mortality. This difference can be explained by the effect from comorbidities and the host immunity. It is essential to take a sex- and age-based approach to SFTS treatment and management.

## Introduction

Severe fever with thrombocytopenia syndrome (SFTS) is an emerging infectious disease, caused by a tick-borne virus, called severe fever with thrombocytopenia syndrome virus (SFTSV), currently also known as *Dabie bandavirus*, which is a novel phlebovirus in the family *Phenuiviridae* of the order *Bunyavirales* (Yu et al., 2011; Kuhn et al., 2020). The disease was identified first in China in 2010 and subsequently in South Korea and Japan in 2013 (Kim et al., 2013; Takahashi et al., 2014). So far, the number of SFTS cases has increased and the geographic distribution has expanded consistently, with the cumulative case numbers attaining 7,721 in China by 2018 (Miao et al., 2020), 1,089 in South Korea by 2019 (Korea Centers for Disease Control and Prevention, 2021)^1^, and 573 in Japan by December 2020 (Japanese National Institute of Infectious Diseases, 2021)^2^, according to the most recent updates. Although the clinical symptoms of SFTS were non-specific, severe complications were reported in critically ill SFTS patients (Yu et al., 2011; Liu et al., 2014), including neurological symptoms, bleeding, and hemophagocytic syndrome, which eventually lead to disseminated intravascular coagulation and multiple organ failures (Gai et al., 2012; Liu et al., 2014), with an estimated case fatality rate (CFR) of 10.5% in China (Miao et al., 2020).

Severe fever with thrombocytopenia syndrome does not impact everyone similarly, with age, sex, and viral load serving as related factors for disease progression and severe SFTS infection (Guo et al., 2016). Substantial evidence supported a greater risk of more severe outcomes in patients with older age (Gai et al., 2012; Zhang et al., 2012; He et al., 2020). In the largest case series study up to date, a higher risk was associated with older age, with an odds ratio (OR) of 1.82 for a 10-year increase (Li et al., 2018).

On the other hand, the association between male and adverse outcomes, including mortality, do not consistently exhibit, which differed across studies (Jung et al., 2019; Wang et al., 2020). More intriguingly, the risk factors that are known to change with sex and age are often interacted, which likely explain the sex differences observed for the mortality risk. Preexisting chronic comorbidity is also an attribute that should be considered, since a large part of SFTS patients were the elderly and frequently afflicted by various underlying conditions which conferred an increased risk of adverse outcomes following infection. Previous studies revealed that adverse outcomes of SFTS patients were associated with comorbidities (Deng et al., 2013; Xu et al., 2018; Zhang et al., 2019), which were specifically identified to be diabetes mellitus (DM), chronic virus hepatitis (CVH), and chronic obstructive pulmonary diseases (COPD) in one study (Zhang et al., 2019). Up to now, no studies in analyzing the risk of SFTS mortality have ever tried to disentangle the effect from age, sex, or comorbidity. Whether sex difference existed for sure or whether both sexes are similarly affected by comorbidities remained unclear.

Here, we examined sex differences in combination with age, comorbidities, and viral load, revealing their complicated interaction in influencing disease severity. We further explored host immunity that was featured by plasma cytokines, chemokines, blood-cell phenotyping, and SFTSV specific antibody in patients, with the aim to reveal the mechanism that underlie the sex preference.

## Materials and Methods

### Study Design and Participants

The retrospective observational study was carried out in the 154 hospital of the Chinese People’s Liberation Army, Xinyang, Henan Province in China, a designated hospital for the sentinel surveillance and treatment for SFTS patients. According to the standard criteria released by China CDC (Huang et al., 2018), a laboratory-confirmed SFTS case was defined as meeting at least one of the following: (1) isolation of SFTSV in cell culture, (2) detection of SFTSV RNA by a molecular method, and (3) seroconversion or ≥4-fold increase of antibody titers between two serum samples collected over 2 weeks apart. Patients with incomplete medical records or laboratory test results or those reported obscure medical history were excluded from the study.

### Data Collection

All patients recruited in the study received the treatment regimens. The demographic (age, sex, and comorbidities) and medication data were collected before treatment. The clinical signs and symptoms, together with laboratory results were recorded on hospital admission and during the treatment. These data were extracted by using a standardized format and entered into an EpiData database. The follow-up interview was performed by telephone to obtain their final outcomes (survival or dead) after discharge from hospital and to ensure that no outcome data was missing.

### Definition of Age Groups and Comorbidities

In the description of baseline information (Table 1), age groups were classified by quantile. In the analyses of viral load and immunological indicators, age groups were divided by median value. For further association analyses, we made grouping on the patients in order to have comparable case numbers among three age groups (<60 years, 60–70 years, and >70 years), which also reflect the impact on the disease outcome. Eight kinds of self-reported comorbidities were classified according to the International Classification of Diseases-10 (ICD-10) and used for the current analysis, including DM, pulmonary tuberculosis (TB), COPD, CVH (HBV and HCV), cerebrovascular diseases (CVD), malignancy, chronic heart diseases (CHD, cardiac heart failure, and coronary heart disease), and hypertension. The corresponding ICD-10 codes were listed in Supplementary Table 1. All the comorbidities were diagnosed before the infectious episode of SFTS, which were acquired by interviewing patients when admission into the hospital.

**TABLE 1:** The risk of age, sex, delay from symptom onset to hospital admission and comorbidity for fatal outcome in SFTS patients.

Characteristics	Total (*N* = 2,938)	Survival (*n* = 2,488)	Fatal (*n* = 450)	Adjusted OR (95%CI)	*P* value
**Age, years, *n* (%)**					
≤50	542 (18.4)	527 (97.2)	15 (2.8)	Reference	
50–60	649 (22.1)	594 (91.5)	55 (8.5)	2.91 (1.62–5.23)	<0.001^#^
60–70	1,011 (34.4)	837 (82.8)	174 (17.2)	6.17 (3.59–10.63)	<0.001^#^
>70	736 (25.1)	530 (72.0)	206 (28.0)	11.43 (6.65–19.66)	<0.001^#^
**Sex, male, *n* (%)**					
Female	1,724 (58.7)	1,489 (86.4)	235 (13.6)	Reference	
Male	1,214 (41.3)	999 (82.3)	215 (17.7)	1.24 (1.00–1.53)	0.048^#^
**Delay from symptom onset to admission, days, n (%)**					
≤5	1,745 (59.4)	1,533 (87.9)	212 (12.1)	Reference	
>5	1,193 (40.6)	955 (80.1)	238 (19.9)	1.56 (1.27–1.93)	<0.001^#^
**Single comorbidity, *n* (%)**					
Non-comorbidity	1,916 (65.2)	1,675 (87.4)	241 (12.6)	Reference	
Any-comorbidity	1,022 (34.8)	813 (79.5)	209 (20.5)	1.48 (1.20–1.83)	<0.001*
DM	186 (6.3)	141 (75.8)	45 (24.2)	2.27 (1.58–3.28)	<0.001*
TB	26 (0.9)	20 (76.9)	6 (23.1)	1.86 (0.73–4.75)	0.196*
COPD	255 (8.7)	197 (77.3)	58 (22.7)	1.84 (1.33–2.56)	<0.001*
CVH	286 (9.7)	226 (79.0)	60 (21.0)	1.88 (1.37–2.59)	<0.001*
CVD	104 (3.5)	84 (80.8)	20 (19.2)	1.50 (0.90–2.50)	0.123*
Malignancy	21 (0.7)	17 (81.0)	4 (19.0)	1.70 (0.56–5.18)	0.351*
CHD	101 (3.4)	82 (81.2)	19 (18.8)	1.61 (0.96–2.72)	0.074*
Hypertension	371 (12.6)	305 (82.2)	66 (17.8)	1.54 (1.14–2.08)	0.005*
**Multiple comorbidities, *n* (%)**					
Zero	1,916 (65.2)	1,675 (87.4)	241 (12.6)	Reference	
One	753 (25.6)	598 (79.4)	155 (20.6)	1.53 (1.22–1.93)	<0.001*
Two	215 (7.3)	174 (80.9)	41 (19.1)	1.32 (0.91–1.93)	0.148*
Three or more	54 (1.8)	41 (75.9)	13 (24.1)	1.39 (0.72–2.70)	0.325*

*OR, odds ratio; CI, confidence interval.*

**P* values were calculated by multivariate logistic regression model.*

*^#^For the age group, the adjusted variables were sex, delay from symptom onset to hospital admission and with any one of comorbidity. For the sex, the adjusted variables were age, delay from symptom onset to hospital admission and with any one of comorbidity. For the delay group, the adjusted variables were age, sex and with any one of comorbidity.*

*Adjusted for age, sex and delay from symptom onset to hospital admission.

### Laboratory Tests

The RNA was extracted from serum samples using QIAamp MinElute Virus Spin Kit (Qiagen, Germantown, MD, United States). The viral load was measured using real-time reverse transcriptase polymerase chain reaction (RT-PCR; Zeng et al., 2017) and expressed as copies/mL. Anti-SFTSV IgM antibodies were detected by using 96-well EIA/RIA Stripwell immunoplates (Corning Costar, NYC). Cytokines and chemokines were measured using Bio-plex Pro Human 27-plex cytokine panel (Bio-Rad Co., Hercules, CA, United States). Phenotypic analysis of peripheral blood mononuclear cells (PBMCs) was detected by multi-parametric flow cytometry. All samples were acquired from Cytoflex flow cytometer and analyzed on CyExpert (Beckman Coulter, Inc., Brea, CA, United States). All laboratory tests were performed according to the manufacturer’s instructions. The detailed protocols or methods were summarized in the Supplementary Material.

### Statistical Analysis

Continuous variables with skewed distribution were summarized as median and interquartile range (IQR), normally distributed variables were expressed as mean and standard deviation (SD). Categorical variables were summarized as frequency and proportion. A non-parametric test, a χ^2^ test, or a Fisher’s exact test was used where appropriate to estimate the differences between groups. The Cochran–Armitage test was used for trend analysis in 2 × k contingency tables.

The association between analyzed variables and fatal outcome was assessed by using multivariate logistic regression model. Adjusted odds ratio (aOR) with 95% confidence intervals (95% CI) was estimated using maximum likelihood methods. The comparison of the inter-group difference of viral loads continuously evaluated over time was performed by generalized estimating equation (GEE). Overall survival was analyzed using Cox regression models, Kaplan–Meier curves, and log-rank tests. Synergy index (SI) that allows assessment of additive interactions was calculated using OR and a 95% CI not across 1 indicated significance. A two-sided *P <* 0.05 was considered statistically significant. All statistical analysis was performed using the R software (version 3.5.3, R Foundation for Statistical Computing, Vienna).

## Results

### Baseline Information of the Patients

From 1 January 2011 to 31 December 2020, a total of 2,938 laboratory-confirmed SFTS patients were admitted to the hospital. The median (IQR) age was 63 (53–71) years old, and 1,724 (58.7%) were female (Table 1 and Supplementary Table 2). Among them, 1,022 (34.8%) had at least one comorbidity, 215 (7.3%) patients presented two comorbidities (hypertension-DM was most frequently observed in 39 patients), and 54 (1.8%) with ≥3 comorbidities (Table 1). Presence of comorbidities was comparable between sex and significantly higher in older patients (*P* = 0.189 and *P* < 0.001; Supplementary Table 2).

### Age- and Sex-Specific CFR

**FIGURE 1:**
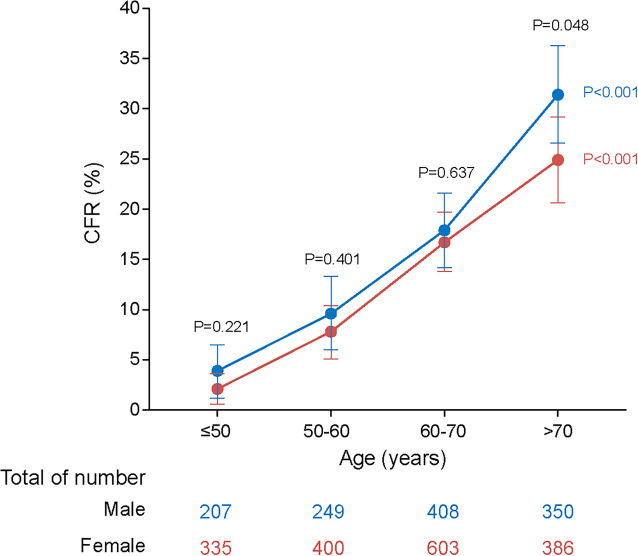
Dynamic profiles of CFRs for males and females across age stratifications in SFTS patients. *P* values were calculated by Chi-square tests. The blue line represents CFRs of males across age stratifications and the red line represents CFRs of females across age stratifications. The error bars indicate 95% CIs. The numbers of males and females across age stratifications were listed on the below. Four age stratifications were classified based on case number: ≤50, 50–60, 60–70, and >70 years. The *P* values colored with blue represent statistical difference among age groups in males. The *P* values colored with red represent statistical difference among age groups in females. The *P* values colored with black represent statistical difference based on age stratification for both male and female patients. As evident from the plot, CFRs rise with increasing age and males are more likely to be affected by aging.

An overall CFR of 15.3% (450/2,938) was obtained, which appeared to be higher in males than in females (17.7% vs. 13.6%, aOR: 1.24, 95% CI: 1.00–1.53; *P* = 0.048) and increased dramatically with age (trend analysis, *P* < 0.001; Table 1 and Figure 1).

Although the outcome was generally worse for males, the magnitude of this sex difference varied among age groups, with significance only observed for those >70 years old (aOR: 1.38, 95% CI: 1.00–1.91; *P* = 0.049; Table 2). On the other hand, although the risk of fatal outcome from older age was observed for both male and female, the magnitude of risk was more pronounced in males. Among females, the ORs for death outcome increased from 3.18 (2.14–4.73) in the 60–70 years old to 5.30 (3.54–7.95) in the >70 years old, while among males, the ORs was elevated from 2.72 (1.74–4.24) to 5.76 (3.75–8.84) with more dramatic extent. Accordingly, the highest CFR was observed among the male patients >70 years old (31.4%; Figure 1 and Table 2).

**TABLE 2:** The risk of CFR based on age, sex and comorbidity stratifications in SFTS patients.

Characteristics	Total (*n* = 2,938)	Survival (*n* = 2,488)	Fatal (*n* = 450)	Adjusted OR (95%CI)	*P* value
**Age stratification, *n* (%)**					
**≤60 years old**					
Female	735 (61.7)	697 (94.8)	38 (5.2)	Reference	
Male	456 (38.3)	424 (93.0)	32 (7.0)	1.38 (0.85–2.26)	0.196*
**60–70 years old**					
Female	603 (59.6)	502 (83.3)	101 (16.7)	Reference	
Male	408 (40.4)	335 (82.1)	73 (17.9)	1.07 (0.77–1.50)	0.683*
**>70 years old**					
Female	386 (52.4)	290 (75.1)	96 (24.9)	Reference	
Male	350 (47.6)	240 (68.6)	110 (31.4)	1.38 (1.00–1.91)	0.049*
**Sex stratification, *n* (%)**					
**Female**					
≤60 years old	735 (42.6)	697 (94.8)	38 (5.2)	Reference	
60–70 years old	603 (35.0)	502 (83.3)	101 (16.7)	3.18 (2.14–4.73)	<0.001*
>70 years old	386 (22.4)	290 (75.1)	96 (24.9)	5.30 (3.54–7.95)	<0.001*
**Male**					
≤60 years old	456 (37.6)	424 (93.0)	32 (7.0)	Reference	
60–70 years old	408 (33.6)	335 (82.1)	73 (17.9)	2.72 (1.74–4.24)	<0.001*
>70 years old	350 (28.8)	240 (68.6)	110 (31.4)	5.76 (3.75–8.84)	<0.001*
**Comorbidity stratification, *n* (%)**					
**Non-comorbidity**					
Female	1,141 (59.6)	1,024 (89.7)	117 (10.3)	Reference	
Male	775 (40.4)	651 (84.0)	124 (16.0)	1.55 (1.17–2.06)	0.002^#^
**Any-comorbidity**					
Female	583 (57.0)	465 (79.8)	118 (20.2)	Reference	
Male	439 (43.0)	348 (79.3)	91 (20.7)	0.94 (0.69–1.29)	0.700^#^
**Comorbidity stratification, *n* (%)**					
**Non-comorbidity**					
≤60 years old	892 (46.6)	850 (95.3)	42 (4.7)	Reference	
60–70 years old	597 (31.2)	513 (85.9)	84 (14.1)	3.11 (2.11–4.59)	<0.001^†^
>70 years old	427 (22.3)	312 (73.1)	115 (26.9)	6.86 (4.69–10.02)	<0.001^†^
**Any-comorbidity**					
≤60 years old	299 (29.3)	271 (90.6)	28 (9.4)	Reference	
60–70 years old	414 (40.5)	324 (78.3)	90 (21.7)	2.61 (1.66–4.12)	<0.001^†^
>70 years old	309 (30.2)	218 (70.6)	91 (29.4)	3.98 (2.51–6.32)	<0.001^†^

**P* values were calculated by multivariate logistic regression model.*

*Adjusted for delay from symptom onset to hospital admission and with any one of comorbidity.

*^#^Adjusted for age and delay from symptom onset to hospital admission.*

*^†^Adjusted for sex and delay from symptom onset to hospital admission.*

### Association Between Comorbidities and Risk of Death

Fatal outcome was reported in 20.5% (209/1022) of the patients with comorbidities, significantly higher than those without comorbidities (12.6%, *P* < 0.001; Table 1). When analyzed separately, four types of comorbidities showed significantly associations with fatal outcome, with aOR (95% CI) estimated to be 2.27 (1.58–3.28) for DM, 1.88 (1.37–2.59) for CVH, 1.84 (1.33–2.56) for COPD, and 1.54 (1.14–2.08) for hypertension, respectively (Table 1). Among patients with comorbidities, the effect from age on increased risk of death was less obvious than among patients without comorbidities. For example, for patients with comorbidities, those of 60–70 years and >70 years were associated with increased risk of death than those aged < 60 years old with aORs (95% CIs) estimated as 2.61 (1.66–4.12) and 3.98 (2.51–6.32), while for patients without comorbidities, the aORs (95% CIs) were estimated as 3.11 (2.11–4.59) and 6.86 (4.69–10.02). Also for patients without comorbidities, males were significantly associated with fatal outcome compared to females (aOR: 1.55, 95% CI: 1.17–2.06; *P* = 0.002; Table 2). Survival analysis likewise demonstrated lower survival probability and shorter survival time in SFTS patients with DM or CVH (both *P* < 0.001; Supplementary Figure 1).

### Age-Stratified Association Between Comorbidities and Death

**FIGURE 2:**
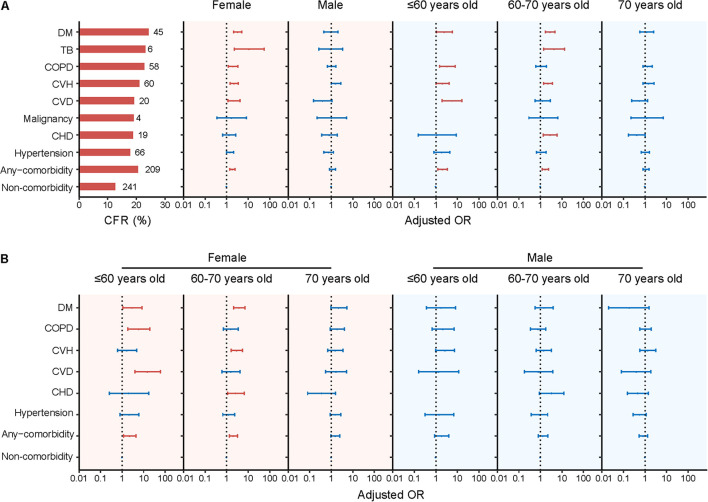
Presence of comorbidity and risk for fatal outcome of SFTS patients stratified by sex or age **(A)** and further stratified by both sex and age **(B)**. The numbers of deaths and CFRs of the SFTS patients with single comorbidity were shown to the left of column in panel **(A)**. Multivariate logistic regression model was performed for association between comorbidity and fatality by adjusting age, sex, and delay between disease onset and hospital admission. Adjusted ORs (aORs) and 95% CIs were presented for each comorbidity. The dots are the aORs and the error bars are the 95% CIs. The red color represents *P* < 0.05 and the blue color represents *P* ≥ 0.05. The dotted line indicates an OR of 1. DM, diabetes mellitus; TB, Pulmonary tuberculosis; COPD, chronic obstructive pulmonary diseases; CVH, chronic viral hepatitis; CVD, cerebrovascular diseases; CHD, chronic heart diseases.

Subgroup analysis revealed that the effect of comorbidity on death was reduced as age increased, i.e., the aOR (95% CI) was 2.07 (1.25–3.42) for patients ≤60 years, decreased to 1.70 (1.22–2.36) for patients aged 60–70 years, and 1.13 (0.81–1.56) for patients aged >70 years (Figure 2 and Supplementary Table 3). Specific comorbidities that were related to fatal outcome also differed among age groups. In patients ≤60 years, four comorbidities showed significantly associations with fatal outcome, with aOR (95% CI) estimated to be 5.68 (1.94–16.57) for CVD, 3.40 (1.47–7.85) for COPD, 2.51 (1.07–5.91) for DM, and 2.04 (1.01–4.13) for CVH, respectively. Among patients aged 60–70 years, DM, CHD, and CVH showed associations with fatal outcome; by contrast, none comorbidities were associated with fatal outcome for patients aged >70 years (Figure 2 and Supplementary Table 3). Resembling these results, survival analysis also displayed lower survival probability and shorter survival time for patients aged >70 years with DM, COPD, CVH, and hypertension, for patients aged 60–70 years with DM and CVH (Supplementary Figure 2). Both findings suggested more aggravating effect from comorbidity within older age group.

The aOR (95% CI) of multiple comorbidities on death also decreased from 3.31 (1.51–7.25) for patients aged ≤60 years to 1.60 (0.97–2.63) for 60–70 years and 0.82 (0.48–1.42) for those aged >70 years when compared with those without comorbidities (Supplementary Figure 3 and Supplementary Table 3).

### Sex-Stratified Association Between Comorbidities and Fatal Outcome

Sex-stratified analysis disclosed significant associations between death and comorbidities for female patients (aOR: 1.87, 95% CI: 1.40–2.49; *P* < 0.001). When analyzed separately, increased risk of fatal outcome was observed for female patients presenting DM, CVH, CVD, or COPD, with aOR (95% CI) estimated as 3.26 (2.08–5.10), 2.21 (1.43–3.42), 2.21 (1.16–4.23), and 1.99 (1.19–3.31), respectively. In contrast, none of the comorbidities was associated with death among male patients (Figure 2 and Supplementary Table 4).

Increased number of comorbidities was related to an increased risk of death, but only among the female patients (aOR: 2.16, 95% CI: 1.42–3.29; *P* < 0.001), not for male patients (Supplementary Figure 3 and Supplementary Table 4).

### Age- and Sex-Stratified Association Between Comorbidities and Fatal Outcome

When both age and sex were disseminated for analysis, the male–female discrepancy remained across each age group in a similar way as that observed for all age groups analyzed together. For example, for female patients, the significant associations between any comorbidity and fatal outcome were observed, however, with aOR (95% CI) decreased from 2.28 (1.16–4.46) in ≤60 years old to 2.06 (1.34–3.18) in 60–70 years old and further to 1.55 (0.97–2.47) in >70 years old. Specific comorbidity that was related to death differed from CVD, COPD, and DM among ≤60 years group; DM, CVH, and CHD among those aged 60–70 years old; and none of the comorbidities for patients aged >70 years. However, for male patients, none of the age group had seen a significant association between fatal outcome and comorbidities no matter if considered as a whole or separately (Figure 2 and Supplementary Tables 4, 5).

In addition, the interactions of age, sex, and comorbidity were evaluated. The SI (95% CI) across 1 indicated that no significant interaction effect on risk of fatal outcome between age and comorbidities in either males or females. The same findings were observed between sex and comorbidities in patients aged ≤60 years and 60–70 years (Supplementary Table 6).

### Age- and Sex-Stratified Association Between Viral Load and Fatal Outcome

Previous studies, including our own study, had identified viral loads as a strong risk factor for fatal outcome of SFTS patients (Gai et al., 2012; Li et al., 2018). We further made age- and sex-specific analysis using viral load as the outcome. By performing GEE model on the serial evaluated viral loads, we observed the association between high viral load and death was stronger in male, with aOR (95%CI) estimated to be 2.15 (1.77–2.62) vs. 1.08 (1.07–1.10) in females. The association between high viral loads and death was decreased as age increased, for example, with ORs decreased from 2.62 (1.73–3.95) in ≤60 years old to 1.10 (1.09–1.12) in the >60 years old (Supplementary Table 7). We also aligned the dynamic pattern of viral load for their difference in sex and age, which showed a significantly high level of viral load in elder age among both females and males (both *P* < 0.001; Supplementary Figure 4), whereas no difference between females and males within each of the evaluate groups (*P* = 0.493 and *P* = 0.714; Supplementary Figure 4).

### The Sex-Related Immune Responses That Underlie the Clinical Phenotype

**FIGURE 3:**
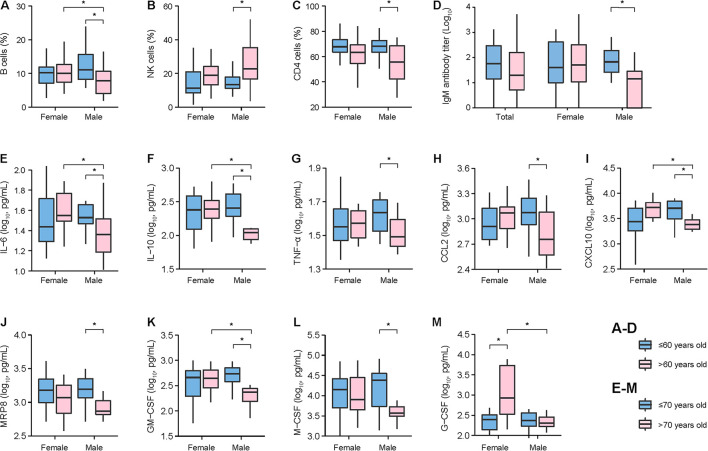
Comparison of cellular immunity **(A–C)**, humoral immunity **(D)**, cytokines, and chemokines **(E–M)** between age groups stratified by sex. *P* values were calculated by Wilcoxon rank sum tests. The age groups were classified by median values. For panels **(A–D)**, two age groups were divided by 60 years. For panels **(E–M)**, two age groups were divided by 70 years. For each evaluation, the median value and the 25th to 75th quantiles were shown. Asterisk (*) means statistical significance of *P* < 0.05 between two age groups.

All the above clinical data indicated that males were more likely to be affected by aging. To determine the mechanism underlying this phenotype, we made further analyses on the sex differences in levels of blood-cell phenotyping, SFTSV-specific antibodies, cytokines, or chemokines under situation of controlling age. Altogether, 194 survived SFTS patients were randomly selected for the test of blood-cell phenotyping and SFTSV-specific antibodies, with their demography highly comparable with the total recruited patients [median age: 62, IQR: 53–70 vs. 63 (53–71) years old; males: 41.8% vs. 41.3%]. A range of indicators, including B cells, natural killer (NK) cells, CD4 cells percentages, and anti-SFTSV IgM antibody level, were detected using the samples that were collected at the early stage of infection. The comparison between age groups was performed for males and females, respectively, which revealed that males >60 years old had significantly decreased percentages of B cells, CD4 cells, lower anti-SFTSV IgM antibody titer, and increased level of NK cells than those ≤60 years old (all *P <* 0.05), while none of these age specific differences was observed in the female (Figure 3 and Supplementary Table 8). The comparison between males and females was performed within two age groups, respectively, showing that the level of B cells, CD4 cells, and anti-SFTSV IgM antibody titer in males were lower than females in aged >60 years old, with significance observed for B cells (Figure 3 and Supplementary Table 9). Altogether, 48 survived SFTS patients (median age: 69, IQR: 62–75; 47.9% males) were measured for 24 cytokines and chemokines on samples that were collected at acute phase of infection. The comparison between two age groups revealed that males aged >70 years had significantly decreased levels of IL-6, IL-10, TNF-α, CCL2, CXCL10, MRP8, GM-CSF, and M-CSF than those aged ≤70 years, while for female patients, only G-CSF demonstrated a significantly higher level in the patients aged >70 years (Figure 3 and Supplementary Table 10). Compared to females, significantly lower levels of IL-6, IL-10, CXCL10, GM-CSF, and G-CSF were detected in males among patients aged >70 years (Figure 3 and Supplementary Table 11).

## Discussion

In previous studies, advanced age, male sex, high viral load, and underlying comorbidities have been proven to be independent risk factors for SFTS adverse outcome and high viral load usually observed in the elderly group (Gai et al., 2012; Zhang et al., 2012, 2019; Deng et al., 2013). In the current study, we further confirmed the complicated interaction among age, sex, viral load, and comorbidities in resulting SFTS adverse outcome. Although the probability of death remained higher in males across all age categories, the magnitude of male-to-female relative risk of death increased as getting old. On the other hand, the differential increased risk by age was more pronounced in males. These findings indicated more important role of aging than sex, and males were more likely to be affected by aging. In addition, viral load showed a lethal effect on SFTS patients according to either sex or age groups, suggesting that viral load was an important factor for predicting adverse outcome in all-age groups and both genders.

Further immunological investigation disclosed that male patients had produced lower plasma levels cytokines, along with poor T-cell and B-cell responses and weak humoral immune response among older group than the younger group, which is more prominent than the age effect from females. Accordingly, males are more likely to have a weaker immune response with aging. There is a well-established notion that older age was commonly considered as a proxy for frailty due to the association between increasing age and frailty (manifested as depressed immunity, increased infections and developed chronic diseases). We could postulate that the key differences in the baseline immune capabilities that differed in males and females of older age might underlie the heightened disease vulnerability owing to an increased age in male than in female.

As has been displayed in previous study, the underlying comorbidities had contributed to the increased fatality, with DM, CVH, and COPD exerting the highest effect (Zhang et al., 2019). By performing data dissemination analysis, we revealed that the effect from preexisting comorbidities was modified by sex and age, with greater effect seen from younger age and female gender. These inequities of underlying disease might be due to the small sample size of the subgroups of patients, for example, the very low case number with preexisting TB. It might also be related to the disproportionate effect on risk of death from both age and sex. It is logical to hypothesize that since male and the elderly are at an increased risk of adverse outcome than their counterpart, the effect from underlying comorbidities was diluted to some extent. In the same way, the death-related effect from age was weak in those with comorbidity, as the effect from aging was diluted by comorbidities. Moreover, the disparity between gender or among age groups that might determine the clinical outcome, such as access to health care, help-seeking behaviors, risk behaviors such as smoking and alcohol consumption, and receiving evidence-based treatment against the preexisting comorbidities, wherein all were potential modifiers for the effect on death (Mauvais-Jarvis et al., 2020). For example, patients with preexisting hypertension regularly take calcium channel blockers as anti-hypertensive medications, which demonstrated to be effective in reducing SFTSV replication and improving outcome (Li et al., 2019). The effect from these confounders is difficult to be separated from the mainstream effect from age and gender.

Sex and age are two of the most relevant factors often associated with health, disease severity, and even mortality (Mauvais-Jarvis et al., 2020). In this context, SFTS might deserve more attention to investigate these differences due to the observations of a higher level of complications and case fatality rates among males (Li et al., 2018). Past studies have suggested a more robust ability among females to control infectious agents (vom Steeg and Klein, 2016). The most recent example was from COVID-19. In one recent study focusing on the sex disparity of COVID-19 patients in immunopathogenic phenotypes exhibited by males and females, Takahashi et al. (2020) illustrated sex heterogeneity in the immune responses to SARS-CoV-2, which could provide novel insights into targetable factors that could mitigate disease. In this context of SFTS, we observed that males had higher NK cells, along with a reduced T-cell and B-cell percentages, as well as decreased levels of several cytokines and chemokines among older group than the younger age. On the other hand, females had shown a minor change of these indicators as age increased, since all of these immunological indicators produced in early stage were associated with disease recovery. For example, poor T-cell response was associated with worse disease outcome following SFTSV infection (Liu et al., 2017). As target for SFTSV infection, peripheral B-cell subsets tended to present a more serious dysregulation in fatal patients (Song et al., 2018). The proportion of NK cells was significantly increased in patients with acute phase and severe SFTS compared to convalescent cases and mild SFTS (Sun et al., 2014). Pronounced cytokine and chemokine levels of IL-6, TNF-α, CCL2, CXCL10, MRP8, GM-CSF, and M-CSF may enhance the inflammation and facilitate the clearance of SFTSV (Sun et al., 2012; Hu et al., 2018). IL-10, as an anti-inflammatory cytokine, can mediate control of excessive immune response and balance immunological reactions (O’Garra and Vieira, 2004). In addition, females may produce a higher level of IgM antibody titer at early SFTSV infection, in line with studies indicating higher levels of serum IgM antibody titer in females (Escobar et al., 1979; Gonzalez-Quintela et al., 2008). All these immunological features provided underlying mechanism to explain why females were less age fragile than males, thus lending to a greater part from comorbidities in influencing the outcome.

The study has several limitations. First, the hospital-based surveillance captured data only from SFTS symptomatic patients who sought medical care and the patients mainly came from Henan province, therefore, the conclusion might need further clarification among asymptomatic patients who failed to seek medical care. Second, the possible coinfection with other tick-borne pathogens was not evaluated, which may alter the CFR-related analysis. Third, data on immune response were not available from all recruited patients and healthy population, especially due to the insufficient number of samples from deceased patients, we were not able to analyze the associations between age, sex, and immunological status with mortality or compare with healthy groups. However, the patients selected for the test of blood-cell phenotyping and SFTSV-specific antibodies showed no disparity from all recruited patients, thus representing unbiased patients of the total. Moreover, the limited sample size hindered further stratified analysis to consider the effect from therapy regimens. Therefore, further study based on a larger sample size and after fully considering the therapy effect should be needed.

In conclusion, the sex difference in the risk of fatal outcome in SFTS patients indeed exist, which was modified in a complicated way by age and comorbidities. These associations with disease outcome might be attributed to the sex difference in the magnitude and efficacy of host immune responses induced by SFTSV infection. This knowledge might enable the public to make truly informed choices about their own disease risk and public policy responses that can be specifically targeted. For example, female patients with SFTS, although at a lower risk of mortality than males, might be at a higher increased risk of death due to underlying health conditions. With currently no proven vaccines or antiviral treatments, aggressive treatment strategies should be applied and the treatment of a broad range of comorbidities should be advocated in high-risk patients. Considering these sex differences in the immune response to SFTSV infection, it is essential to take a sex- and age-based approach to the overall treatment and management. Further elucidation of sex differences in the immune response to SFTSV infection that may play a role in determining the outcome has the potential to provide therapeutic insights and contribute to precision medical interventions in the future.

## Data Availability Statement

The raw data supporting the conclusions of this article will be made available by the authors, without undue reservation.

## Ethics Statement

The studies involving human participants were reviewed and approved by the Ethics Committees from the 154 hospital of the Chinese People’s Liberation Army. The patients/participants provided their written informed consent to participate in this study. Written informed consent was obtained from the individual(s) for the publication of any potentially identifiable images or data included in this article.

## Author Contributions

WL, L-QF, Q-BL, and JZ provided conception and designed the study. NC, CY, JD, S-MR, and L-ZL collected the epidemiological data and conducted laboratory tests. WL, Q-BL, JZ, and YY cleaned, analyzed, and interpreted the data. WL, L-QF, X-AZ, and Z-DY provided administrative, technical, or logistic support. WL, JZ, and YY drafted the manuscript. WL, Q-BL, HL, and L-QF provided critical revision of the article for important intellectual content. JZ, Q-BL, and WL had access to and verified the underlying data. All authors read and approved the final report.

## Conflict of Interest

The authors declare that the research was conducted in the absence of any commercial or financial relationships that could be construed as a potential conflict of interest.

## Publisher’s Note

All claims expressed in this article are solely those of the authors and do not necessarily represent those of their affiliated organizations, or those of the publisher, the editors and the reviewers. Any product that may be evaluated in this article, or claim that may be made by its manufacturer, is not guaranteed or endorsed by the publisher.

## Footnotes

1
https://is.kdca.go.kr

2
https://www.niid.go.jp

## Supplementary Material

Supplementary Figure 1 Kaplan–Meier survival curves for specific comorbidity. *P* value was calculated using multiple Cox regression model, adjusting for age, sex, and delay from symptom onset to hospital admission. The numbers of patients without comorbidity and with specific comorbidity across days from symptom onset were listed on the below. The blue lines and numbers indicate non-comorbidity. The red lines and numbers indicate specific comorbidity.

Supplementary Figure 2 Kaplan–Meier survival curves based on age stratification for specific comorbidity. *P* value was calculated using multiple Cox regression model, adjusting for sex and delay from symptom onset to hospital admission. The numbers of patients age ≤60 years, 60–70 years, and >70 years across days from symptom onset were listed on the below. The blue lines and numbers indicate age ≤60 years, the yellow lines and numbers indicate 60–70 years, and the red lines and numbers indicate >70 years.

Supplementary Figure 3 Presence of multiple coexisting comorbidities and risk for fatal outcome of SFTS patients stratified by sex **(A)** or age **(B)**. The numbers of deaths and CFRs of the SFTS patients with multiple coexisting comorbidities were shown to the left of column in panel **(A)**. Multivariate logistic regression model was performed for association between comorbidity and fatality by adjusting age, sex, and delay from symptom onset to hospital admission. Adjusted ORs (aORs) and 95% CIs were presented for multiple coexisting comorbidities. The dots are the aORs and the error bars are the 95% CIs. The red color represents *P* < 0.05 and the blue color represents *P* ≥ 0.05. The dotted line indicates an OR of 1.

Supplementary Figure 4 Kinetics of viral load stratified by sex and age in SFTS patients. *P* values were calculated by generalized estimating equation model. The adjusted variables were delay from symptom onset to hospital admission and with any one of comorbidity. Viral load was measured as log10 copies/mL. Age groups were classified by median value. The dots and the error bars denote medians and IQRs. *P* < 0.05 indicates statistically significant.
