# Supplementary material for: Sex Differences in Case Fatality Rate of Patients With Severe Fever With Thrombocytopenia Syndrome
Source: Front Microbiol. 2021 Oct 14;12:738808. doi: 10.3389/fmicb.2021.738808 (PMC8552034; doi:10.3389/fmicb.2021.738808)
Supplement: Supplementary file 1 [file Data_Sheet_1.docx]

**Supplementary Contents**

**Materials and Methods**

**Supplemental Tables**

**Table S1.** The ICD-10 Code for comorbidities included in the current study.

**Table S2.** Demographics and clinical characteristics of SFTS patients based on existence of comorbidity.

**Table S3.** The age stratified risk of comorbidity for fatal outcome in SFTS patients.

**Table S4.** The sex stratified risk of comorbidity for fatal outcome in SFTS patients.

**Table S5.** Age-specific risk of comorbidity for fatal outcome stratified by sex in SFTS patients.

**Table S6.** The interactions of age, sex and comorbidity for the risk of fatal outcome in SFTS patients.

**Table S7.** Theage and sex stratified risk of viral load for fatal outcome in SFTS patients.

**Table S8.** Comparison of cellular immunity and humoral immunity between age groups stratified by sex.

**Table S9.** Comparison of cellular immunity and humoral immunity between sex stratified by age.

**Table S10.** Comparison of cytokines and chemokines between age groups stratified by sex.

**Table S11.** Comparison of cytokines and chemokines between sex stratified by age.

**Materials and Methods**

**Detection and quantitation of SFTSV**

The SFTSV RNA was extracted from 200 μl serum samples using the QIAamp MinElute Virus Spin Kit (Qiagen, Germantown, MD) according to the manufacturer’s instructions. One step Primer Script Reverse Transcription-polymerase Chain Reaction (RT-PCR) Kit (TaKaRa Bio, Shiga, Japan) was used for SFTSV detection in a volume of 20 µl containing 10 µl of One Step RT-PCR Buffer (2×), 0.4 µl of TaKaRa Ex Taq HS (5 U/µl) and 0.4 µl of PrimeScriptTM RT Enzyme Mix II, 1 µl of PCR primer mix (20 µM of sense and antisense each) and 0.5µl of Probe (10 µM), total RNA 2 µl and RNase free dH_2_O (5.7 µl). PCR was carried with one cycle of 42ºC for 5 min and 95ºC for 10 sec, followed by 40 cycles of 95ºC for 5 sec and 55ºC for 20 sec in a LightCycler Real Time PCR (Roche Diagnostics, Germany). The real-time PCR primers and probe were targeted at the S-segment of the SFTSV. The sequences were as follows: forward: TTCACAGCAGCATGGAGAGG; reverse: GATGCCTTCACCAAGACTATCAATG; Probe: AACTTCTGTCTTGCTGGCTCCGC.

The quantification of SFTSV was measured using real-time RT-PCR (Roche Diagnostics, Germany) targeting the same gene segments according to the protocol. Standard curves or absolute RNA quantification were included in every assay and were generated by using RNA transcripts produced by in vitro transcription of cDNA that included the real time qPCR assay amplicons. All samples were quantified in at least duplicate wells. Levels of SFTSV RNA concentrations were expressed as copies/mL.

**Detection of SFTSV specific IgM antibody**

Anti-SFTSV IgM antibodies were detected by using the 96-well EIA/RIA Stripwell immunoplates (Corning Costar, NYC) according to the manufacturer’s instructions. In the initial screening, an undiluted serum sample was used to determine whether the sample was positive for antibodies against SFTSV. Positive serum samples were diluted starting at 1:10 and added to the wells. Plates were incubated for 30 min at 37°C and washed 5 times. Antihuman IgM conjugates were added. After incubation and washing 5 times as described above, 100μL TMB (Tetramethylbenzidine) substrate was added to the wells to promote the color reaction. Plates were incubated for 10 min at room temperature and reaction was stopped with 100 μl 1 M H2SO4 stopping solution. Absorbance value was read at 450nm and reported as optical density (OD) values.

**Detection of cytokines and chemokines**

Cytokines and chemokines were measured from the acute serum samples using the Bio-plex Pro Human 27-plex cytokine panel (Bio-Rad Co., Hercules, CA) on the Bio-Plex platform following workflow. 24 cytokines/chemokines levels including IL-1β, IL-6, Il-8, IL-10, Il-12p70, IL-18, TNF-α, IFN-γ, CCL2, CCL5, CXCL10-VEGF-A, P-selectin, MMP-9, Angiopoieitin-1, MRP8, Angiopoietin-2, E-selectin, GM-CSF, M-CSF, G-CSF, VCAM-1, ICAM-1 and CD40 Ligend were evaluated. First, 50μl of beads were added to each well in 96-well microplates. After one wash, 50μl of standard or sample was added to each well and incubated for 1 hour with shaking. After a wash with wash buffer, 25μl of biotin-labelled detection antibody was added, and the plates were incubated for 30min with shaking. After a wash, 50μl of streptavidin-PE was added, and the plates were incubated for 10min with shaking. Finally, the beads in each well were resuspended in 125μl of assay buffer, and the entire plate was read on a Bio-Plex system.

**Detection of peripheral blood mononuclear cells (PBMCs)**

Phenotypic analysis of PBMCs were detected by multi-parametric flow cytometry. All samples were acquired from Cytoflex flow cytometer and analyzed on CyExpert software (Beckman Coulter, Inc., Brea, CA). PBMCs were blocked with Human TruStain FcX (Biolegend, San Diego, CA) for 10min at RT, and then stained with surface mAbs for 25 min at 4°C with saturating concentrations of mAbs.

Sampleswere washed twice with PBS after cell surface marker staining, then remaining cells were fixed and permeabilized using Fixation/Permeabilization solution kits (BD Biosciences) for 25min at 4 °C. These samples were washed with BD Perm/Wash Buffer and incubated with specific mAbs toward intracellular molecule for 30 min at 4°C. After incubation, these samples were washed again with BD Perm/Wash Buffer and resuspended in PBS for the test by Cytoflex flow cytometer. CD45+ cells were used to identify leukocytes, the cellular fractions were identified as CD3-CD19+ B cells, CD3+ T cells, CD3-CD56+ NK cells.

**Table S1. The ICD-10 Code for comorbidities included in the current study.**

| **Number** | **ICD-10 code** | **Comorbidity** |
| --- | --- | --- |
| 1 | E14.800 | diabetes mellitus (DM, both type I and type II) |
| 2 | A15-A19 | pulmonary tuberculosis (TB) |
| 3 | J44.901 | chronic obstructive pulmonary diseases (COPD) |
| 4 | B18.951 | chronic viral hepatitis (CVH, HBV and HCV) |
| 5 | I67.800 | cerebrovascular diseases (CVD) |
| 6 | C00-D48 | malignancy |
| 7 | I50, I25.101 | chronic heart diseases (CHD, cardiac heart failure and coronary heart disease) |
| 8 | I10.X02 | hypertension |

**Table S2. Demographics and clinical characteristics of SFTS patients based on existence of comorbidity.**

| **Characteristics** | **Total**  **(N=2938)** | **Comorbidity** | | **P value** |
| --- | --- | --- | --- | --- |
|  |  | **No (n=1916)** | **Yes (n=1022)** |  |
| **Age, years, median (IQR)** | 63 (53-71) | 62 (51-70) | 66 (59-72) | <0.001 |
| ≤50 | 542 (18.4) | 455 (83.9) | 87 (16.1) | <0.001 |
| 50-60 | 649 (22.1) | 437 (67.3) | 212 (32.7) |  |
| 60-70 | 1011 (34.4) | 597 (59.1) | 414 (40.9) |  |
| >70 | 736 (25.1) | 427 (58.0) | 309 (42.0) |  |
| **Sex, n (%)** |  |  |  |  |
| Female | 1724 (58.7) | 1141 (66.2) | 583 (33.8) | 0.189 |
| Male | 1214 (41.3) | 775 (63.8) | 439 (36.2) |  |
| **Interval period, days, median (IQR)** |  |  |  |  |
| From symptom onset to admission | 5 (4-7) | 5 (4-7) | 5 (4-7) | 0.079 |
| Duration of hospital stay | 8 (5-10) | 8 (5-10) | 7 (5-10) | 0.040 |
| Duration of clinical course^*^ | 13 (10-15) | 13 (10-15) | 13 (10-15) | 0.199 |
| **Clinical manifestations, n (%)** |  |  |  |  |
| Fever | 2927 (99.6) | 1910 (99.7) | 1017 (99.5) | 0.669 |
| Chill | 368 (12.5) | 226 (11.8) | 142 (13.9) | 0.102 |
| Headache | 407 (13.9) | 236 (12.3) | 171 (16.7) | 0.001 |
| Dizziness | 587 (20.0) | 355 (18.5) | 232 (22.7) | 0.007 |
| Feeble | 2772 (94.3) | 1801 (94.0) | 971 (95.0) | 0.258 |
| Myalgia | 2363 (80.4) | 1543 (80.5) | 820 (80.2) | 0.846 |
| Lymphadenectasis | 1608 (54.7) | 1048 (54.7) | 560 (54.8) | 0.960 |
| Sclera | 8 (0.3) | 4 (0.2) | 4 (0.4) | 0.594 |
| Rash | 32 (1.1) | 26 (1.4) | 6 (0.6) | 0.055 |
| **Bleeding symptoms, n (%)** | 1048 (35.7) | 633 (33.0) | 415 (40.6) | <0.001 |
| Bloodystool | 179 (6.1) | 122 (6.4) | 57 (5.6) | 0.394 |
| Gingival bleeding | 288 (9.8) | 159 (8.3) | 129 (12.6) | <0.001 |
| Hemoptysis | 112 (3.8) | 60 (3.1) | 52 (5.1) | 0.008 |
| Haematemesis | 64 (2.2) | 42 (2.2) | 22 (2.2) | 0.944 |
| Petechia | 666 (22.7) | 396 (20.7) | 270 (26.4) | <0.001 |
| Epistaxis | 28 (1.0) | 14 (0.7) | 14 (1.4) | 0.089 |
| Macroscopic haematuria | 121 (4.1) | 63 (3.3) | 58 (5.7) | 0.002 |
| Conjunctiva hemorrhage | 11 (0.4) | 8 (0.4) | 3 (0.3) | 0.836 |
| **Respiratory symptoms, n (%)** |  |  |  |  |
| Cough | 1482 (50.4) | 893 (46.6) | 589 (57.6) | <0.001 |
| Sputum | 1140 (38.8) | 667 (34.8) | 473 (46.3) | <0.001 |
| Dyspnea | 229 (7.8) | 126 (6.6) | 103 (10.1) | 0.001 |
| **Gastrointestinal symptoms, n (%)** |  |  |  |  |
| Anorexia | 2326 (79.2) | 1471 (76.8) | 855 (83.7) | <0.001 |
| Nausea | 2171 (73.9) | 1405 (73.3) | 766 (75.0) | 0.341 |
| Vomit | 1146 (39.0) | 707 (36.9) | 439 (43.0) | 0.001 |
| Abdominal | 261 (8.9) | 157 (8.2) | 104 (10.2) | 0.081 |
| Bloating | 226 (7.7) | 117 (6.1) | 109 (10.7) | <0.001 |
| Diarrhea | 880 (30.0) | 567 (29.6) | 313 (30.6) | 0.560 |

Continuous variables with skewed distribution were summarized as median and interquartile range (IQR).Categorical variables were summarized as frequency and proportion.P values were calculated by Wilcoxon rank sum testsandChi-square testswhere appropriate to estimate the differences between groups.

^*^Clinical course referred to days from symptom onset to discharge or death.

**Table S3. The age stratified risk of comorbidity forfatal outcome in SFTS patients.**

| **Comorbidity** | **Survival (n=2488)** | **Fatal (n=450)** | **Adjusted OR (95% CI)^*^** | ***P* value** |
| --- | --- | --- | --- | --- |
| **≤60 years old,n (%)** | **1121 (94.1)** | **70 (5.9)** |  |  |
| Non-comorbidity | 850 (95.3) | 42 (4.7) | Reference |  |
| Any-comorbidity | 271 (90.6) | 28 (9.4) | 2.07 (1.25-3.42) | 0.005 |
| **Single comorbidity** |  |  |  |  |
| DM | 55 (88.7) | 7 (11.3) | 2.51 (1.07-5.91) | 0.035 |
| COPD | 42 (84.0) | 8 (16.0) | 3.40 (1.47-7.85) | 0.004 |
| CVH | 108 (90.8) | 11 (9.2) | 2.04 (1.01-4.13) | 0.047 |
| CVD | 16 (76.2) | 5 (23.8) | 5.68 (1.94-16.57) | 0.001 |
| CHD | 17 (94.4) | 1 (5.6) | 1.14 (0.15-8.90) | 0.901 |
| Hypertension | 80 (92.0) | 7 (8.0) | 1.90 (0.82-4.40) | 0.136 |
| **Multiple comorbidities** |  |  |  |  |
| One | 219 (92.0) | 19 (8.0) | 1.76 (0.99-3.10) | 0.051 |
| Two or more | 52 (85.2) | 9 (14.8) | 3.31 (1.51-7.25) | 0.003 |
| **60-70 years old,n (%)** | **837 (82.8)** | **174 (17.2)** |  |  |
| Non-comorbidity | 513 (85.9) | 84 (14.1) | Reference |  |
| Any-comorbidity | 324 (78.3) | 90 (21.7) | 1.70 (1.22-2.36) | 0.002 |
| **Single comorbidity** |  |  |  |  |
| DM | 60 (69.0) | 27 (31.0) | 2.86 (1.71-4.79) | <0.001 |
| COPD | 92 (84.4) | 17 (15.6) | 1.07 (0.61-1.91) | 0.808 |
| CVH | 79 (73.1) | 29 (26.9) | 2.26 (1.39-3.68) | 0.001 |
| CVD | 38 (82.6) | 8 (17.4) | 1.28 (0.57-2.85) | 0.552 |
| CHD | 27 (69.2) | 12 (30.8) | 2.86 (1.37-5.95) | 0.005 |
| Hypertension | 124 (84.9) | 22 (15.1) | 1.10 (0.66-1.85) | 0.705 |
| **Multiple comorbidities** |  |  |  |  |
| One | 227 (77.7) | 65 (22.3) | 1.74 (1.21-2.50) | 0.003 |
| Two or more | 97 (79.5) | 25 (20.5) | 1.60 (0.97-2.63) | 0.067 |
| **>70 years old,n (%)** | **530 (72.0)** | **206 (28.0)** |  |  |
| Non-comorbidity | 312 (73.1) | 115 (26.9) | Reference |  |
| Any-comorbidity | 218 (70.6) | 91 (29.4) | 1.13 (0.81-1.56) | 0.478 |
| **Single comorbidity** |  |  |  |  |
| DM | 26 (70.3) | 11 (29.7) | 1.21 (0.57-2.56) | 0.613 |
| COPD | 63 (65.6) | 33 (34.4) | 1.32 (0.81-2.13) | 0.261 |
| CVH | 39 (66.1) | 20 (33.9) | 1.46 (0.81-2.62) | 0.211 |
| CVD | 30 (81.1) | 7 (18.9) | 0.57 (0.24-1.34) | 0.196 |
| CHD | 38 (86.4) | 6 (13.6) | 0.42 (0.17-1.03) | 0.057 |
| Hypertension | 101 (73.2) | 37 (26.8) | 1.02 (0.66-1.58) | 0.925 |
| **Multiple comorbidities** |  |  |  |  |
| One | 152 (68.2) | 71 (31.8) | 1.26 (0.88-1.80) | 0.206 |
| Two or more | 66 (76.7) | 20 (23.3) | 0.82 (0.48-1.42) | 0.480 |

P values were calculated by multivariate logistic regression model.

^*^ For the age stratification, the reference group was patients without comorbidity, the adjusted variables were sex and delay from symptom onset to hospital admission. TB and malignancy were excluded from the age stratified analysis due to small case number.

**Table S4. The sex stratified risk of comorbidity for fatal outcome in SFTS patients.**

| **Comorbidity** | **Survival (n=2488)** | **Fatal (n=450)** | **Adjusted OR (95% CI)^*^** | ***P* value** |
| --- | --- | --- | --- | --- |
| **Female, n (%)** | **1489 (86.4)** | **235 (13.6)** |  |  |
| Non-comorbidity | 1024 (89.7) | 117 (10.3) | Reference |  |
| Any-comorbidity | 465 (79.8) | 118 (20.2) | 1.87 (1.40-2.49) | <0.001 |
| **Single comorbidity** |  |  |  |  |
| DM | 93 (72.1) | 36 (27.9) | 3.26 (2.08-5.10) | <0.001 |
| COPD | 77 (75.5) | 25 (24.5) | 1.99 (1.19-3.31) | 0.008 |
| CVH | 138 (79.8) | 35 (20.2) | 2.21 (1.43-3.42) | <0.001 |
| CVD | 41 (73.2) | 15 (26.8) | 2.21 (1.16-4.23) | 0.016 |
| CHD | 52 (82.5) | 11 (17.5) | 1.30 (0.64-2.61) | 0.467 |
| Hypertension | 198 (81.5) | 45 (18.5) | 1.44 (0.97-2.13) | 0.069 |
| **Multiple comorbidities** |  |  |  |  |
| One | 342 (81.4) | 78 (18.6) | 1.75 (1.26-2.41) | <0.001 |
| Two or more | 123 (75.5) | 40 (24.5) | 2.16 (1.42-3.29) | <0.001 |
| **Male, n (%)** | **999 (82.3)** | **215 (17.7)** |  |  |
| Non-comorbidity | 651 (84.0) | 124 (16.0) | Reference |  |
| Any-comorbidity | 348 (79.3) | 91 (20.7) | 1.13 (0.83-1.55) | 0.436 |
| **Single comorbidity** |  |  |  |  |
| DM | 48 (84.2) | 9 (15.8) | 0.96 (0.45-2.06) | 0.921 |
| COPD | 120 (78.4) | 33 (21.6) | 1.05 (0.67-1.65) | 0.819 |
| CVH | 88 (77.9) | 25 (22.1) | 1.65 (0.99-2.75) | 0.054 |
| CVD | 43 (89.6) | 5 (10.4) | 0.40 (0.15-1.06) | 0.066 |
| CHD | 30 (78.9) | 8 (21.1) | 0.83 (0.36-1.91) | 0.666 |
| Hypertension | 107 (83.6) | 21 (16.4) | 0.76 (0.45-1.28) | 0.303 |
| **Multiple comorbidities** |  |  |  |  |
| One | 256 (76.9) | 77 (23.1) | 1.33 (0.95-1.85) | 0.095 |
| Two or more | 92 (86.8) | 14 (13.2) | 0.62 (0.34-1.15) | 0.128 |

P values were calculated by multivariate logistic regression model.

^*^ For the sex stratification, the reference group was patients without comorbidity, the adjusted variables were age and delay from symptom onset to hospital admission. TB and malignancy were excluded from the sex stratified analysis due to small case number.

**Table S5. Age-specific risk of comorbidity for fatal outcome stratified by sex in SFTS patients.**

| **Comorbidity** | **Total** | **≤60 years old** | | **60-70 years old** | | **>70 years old** | |
| --- | --- | --- | --- | --- | --- | --- | --- |
|  | **N (CFR%)** | **n (CFR%)** | **Adjusted OR (95% CI)**^*^ | **n (CFR%)** | **Adjusted OR (95% CI)**^*^ | **n (CFR%)** | **Adjusted OR (95% CI)**^*^ |
| **All patients** |  |  |  |  |  |  |  |
| Non-comorbidity | 1916 (12.6) | 892 (4.7) | Reference | 597 (14.1) | Reference | 427 (26.9) | Reference |
| Any-comorbidity | 1022 (20.5) | 299 (9.4) | 2.07 (1.25-3.41)^#^ | 414 (21.7) | 1.70 (1.22-2.36)^#^ | 309 (29.4) | 1.13 (0.81-1.56) |
| **Single comorbidity** |  |  |  |  |  |  |  |
| DM | 186 (24.2) | 62 (11.3) | 2.51 (1.07-5.91)^#^ | 87 (31.0) | 2.86 (1.71-4.79)^#^ | 37 (29.7) | 1.21 (0.57-2.56) |
| COPD | 255 (22.7) | 50 (16.0) | 3.40 (1.47-7.85)^#^ | 109 (15.6) | 1.07 (0.61-1.91) | 96 (34.4) | 1.32 (0.81-2.13) |
| CVH | 286 (21.0) | 119 (9.2) | 2.04 (1.01-4.13)^#^ | 108 (26.9) | 2.26 (1.39-3.68)^#^ | 59 (33.9) | 1.46 (0.81-2.62) |
| CVD | 104 (19.2) | 21 (23.8) | 5.68 (1.94-16.57)^#^ | 46 (17.4) | 1.28 (0.57-2.85) | 37 (18.9) | 0.57 (0.24-1.34) |
| CHD | 101 (18.8) | 18 (5.6) | 1.14 (0.15-8.90) | 39 (30.8) | 2.86 (1.37-5.95)^#^ | 44 (13.6) | 0.42 (0.17-1.03) |
| Hypertension | 371 (17.8) | 87 (8.0) | 1.90 (0.82-4.40) | 146 (15.1) | 1.10 (0.66-1.85) | 138 (26.8) | 1.02 (0.66-1.58) |
| **Female** |  |  |  |  |  |  |  |
| Non-comorbidity | 1141 (10.3) | 552 (4.0) | Reference | 358 (12.6) | Reference | 231 (21.6) | Reference |
| Any-comorbidity | 583 (20.2) | 183 (8.7) | 2.28 (1.16-4.46)^#^ | 245 (22.9) | 2.06 (1.34-3.18)^#^ | 155 (29.7) | 1.55 (0.97-2.47) |
| **Single comorbidity** |  |  |  |  |  |  |  |
| DM | 129 (27.9) | 43 (11.6) | 3.02 (1.08-8.48)^#^ | 60 (35.0) | 3.86 (2.07-7.18)^#^ | 26 (38.5) | 2.25 (0.96-5.27) |
| COPD | 102 (24.5) | 20 (20.0) | 5.98 (1.83-19.59)^#^ | 47 (19.1) | 1.56 (0.70-3.46) | 35 (34.3) | 1.92 (0.89-4.15) |
| CVH | 173 (20.2) | 72 (6.9) | 1.70 (0.62-4.68) | 67 (29.9) | 2.98 (1.61-5.51)^#^ | 34 (29.4) | 1.55 (0.69-3.46) |
| CVD | 56 (26.8) | 10 (40.0) | 15.48 (4.01-59.73)^#^ | 31 (19.4) | 1.58 (0.61-4.10) | 15 (33.3) | 1.68 (0.54-5.21) |
| CHD | 63 (17.5) | 12 (8.3) | 2.10 (0.26-17.06) | 29 (27.6) | 2.68 (1.10-6.51)^#^ | 22 (9.1) | 0.36 (0.08-1.59) |
| Hypertension | 243 (18.5) | 61 (8.2) | 2.18 (0.79-6.00) | 99 (15.2) | 1.24 (0.66-2.34) | 83 (30.1) | 1.57 (0.89-2.76) |
| **Male** |  |  |  |  |  |  |  |
| Non-comorbidity | 775 (16.0) | 340 (5.9) | Reference | 239 (16.3) | Reference | 196 (33.2) | Reference |
| Any-comorbidity | 439 (20.7) | 116 (10.3) | 1.86 (0.87-3.97) | 169 (20.1) | 1.31 (0.78-2.19) | 154 (29.2) | 0.83 (0.53-1.32) |
| **Single comorbidity** |  |  |  |  |  |  |  |
| DM | 57 (15.8) | 19 (10.5) | 1.77 (0.37-8.34) | 27 (22.2) | 1.49 (0.56-3.95) | 11 (9.1) | 0.19 (0.02-1.55) |
| COPD | 153 (21.6) | 30 (13.3) | 2.16 (0.67-6.95) | 62 (12.9) | 0.78 (0.34-1.79) | 61 (34.4) | 1.05 (0.57-1.94) |
| CVH | 113 (22.1) | 47 (12.8) | 2.68 (0.99-7.29) | 41 (22.0) | 1.45 (0.64-3.28) | 25 (40.0) | 1.35 (0.58-3.19) |
| CVD | 48 (10.4) | 11 (9.1) | 1.34 (0.16-11.42) | 15 (13.3) | 0.82 (0.18-3.77) | 22 (9.1) | 0.40 (0.08-1.86) |
| CHD | 38 (21.1) | 6 (0) | - | 10 (40.0) | 3.31 (0.88-12.41) | 22 (18.2) | 0.47 (0.15-1.44) |
| Hypertension | 128 (16.4) | 26 (7.7) | 1.45 (0.31-6.75) | 47 (14.9) | 0.90 (0.37-2.17) | 55 (21.8) | 0.57 (0.28-1.15) |

n, the number of patients with the comorbidity in the stratification. CFR%, the case fatalityrate of patents with the comorbidity in the stratification. TB and malignancy were excluded from the age- and sex- stratified analysis due to small case number.

P values were calculated by multivariate logistic regression model.

^*^ Adjusted for delay from symptom onset to hospital admission.

^#^ P value <0.05.

**Table S6. The interactions of age, sex and comorbidity for the risk of fatal outcome in SFTS patients.**

| **Coexisting factors** | **Adjusted OR (95% CI)^*^** | ***P* value** | **SI (95% CI)^#^** |
| --- | --- | --- | --- |
| **Sex stratification** |  |  |  |
| **Female** |  |  |  |
| ≤60years old & Without comorbidity | Reference |  |  |
| ≤60years old & With comorbidity | 2.27 (1.17-4.44) | 0.016 |  |
| 60-70years old & Without comorbidity | 3.36 (1.97-5.71) | <0.001 |  |
| 60-70years old & With comorbidity | 6.92 (4.1-11.68) | <0.001 | 1.63 (0.91-2.92) |
| **Male** |  |  |  |
| ≤60years old & Without comorbidity | Reference |  |  |
| ≤60years old & With comorbidity | 1.85 (0.87-3.93) | 0.111 |  |
| 60-70years old & Without comorbidity | 2.92 (1.64-5.18) | <0.001 |  |
| 60-70years old & With comorbidity | 3.84 (2.12-6.94) | <0.001 | 1.03 (0.47-2.21) |
| **Age stratification** |  |  |  |
| **≤60 years old** |  |  |  |
| Female & Without comorbidity | Reference |  |  |
| Female & With comorbidity | 2.27 (1.16-4.47) | 0.017 |  |
| Male & Without comorbidity | 1.51 (0.80-2.83) | 0.202 |  |
| Male & With comorbidity | 2.78 (1.32-5.85) | 0.007 | 1.00 (0.29-3.45) |
| **60-70 years old** |  |  |  |
| Female & Without comorbidity | Reference |  |  |
| Female & With comorbidity | 2.06 (1.34-3.18) | 0.001 |  |
| Male & Without comorbidity | 1.34 (0.84-2.13) | 0.219 |  |
| Male & With comorbidity | 1.74 (1.06-2.84) | 0.027 | 0.53 (0.18-1.50) |

Comorbidities had no effect on increasing risk of fatal outcome among patients >70 years old, so the results of age >70 years old were not shown.

^*^ Adjusted for delay from symptom onset to hospital admission.

^#^ SI indicates synergy index that allows assessment of binary interactions. The synergy index (SI) greater than 1 means positive interaction or more than additivity.

**Table S7. Theage and sex stratified risk of viral load for fatal outcome in SFTS patients.**

| **Characteristics** | **Adjusted OR (95%CI)** | **P value** |
| --- | --- | --- |
| **Sex** |  |  |
| Female | 1.08 (1.07-1.10) | <0.001^#^ |
| Male | 2.15 (1.77-2.62) | <0.001^#^ |
| **Age** |  |  |
| ≤60 years old | 2.62 (1.73-3.95) | <0.001^#^ |
| >60 years old | 1.10 (1.09-1.12) | <0.001^#^ |
| **Female** |  |  |
| ≤60 years old | 1.26 (1.13-1.41) | <0.001^*^ |
| >60 years old | 1.09 (1.07-1.11) | <0.001^*^ |
| **Male** |  |  |
| ≤60 years old | 2.12 (1.13-3.97) | 0.019^*^ |
| >60 years old | 1.12 (1.09-1.15) | <0.001^*^ |

P values were calculated by generalized estimating equation model. Age groups were classified by median value.

^#^ For the sex, the adjusted variables were age, delay from symptom onset to hospital admission and with any one of comorbidity. For the age group, the adjusted variables were sex, delay from symptom onset to hospital admission and with any one of comorbidity.

^*^ Adjusted for delay from symptom onset to admission and with any one of comorbidity.

**Table S8. Comparison of cellular immunity and humoral immunitybetween age groups stratified by sex.**

| **Parameters** | **Total**  **(N=113)** | **Female** | | | **Total**  **(N=81)** | **Male** | | |
| --- | --- | --- | --- | --- | --- | --- | --- | --- |
|  |  | **≤60 years old**  **(n=52)** | **>60 years old**  **(n=61)** | ***P* value** |  | **≤60 years old**  **(n=34)** | **>60 years old**  **(n=47)** | ***P* value** |
| **B cells (%)** | 10.10 (7.09-12.40) | 10.18 (7.11-11.86) | 10.01 (7.41-12.63) | 0.951 | 9.38 (6.88-14.14) | 11.07 (8.23-15.61) | 7.85 (4.07-10.68) | 0.045 |
| **NK cells (%)** | 17.39 (11.21-23.48) | 11.23 (8.41-20.97) | 18.89 (13.25-24.10) | 0.125 | 18.44 (13.19-27.10) | 13.39 (11.23-17.76) | 22.81 (17.10-38.94) | <0.001 |
| **CD4 cells (%)** | 65.15 (56.95-71.67) | 67.70 (63.00-73.10) | 63.33 (54.56-69.24) | 0.108 | 62.95 (52.80-71.38) | 68.28 (63.36-72.66) | 55.60 (42.20-68.55) | <0.001 |
| **IgM titer** | 1.60 (1.00-2.51) | 1.60 (1.00-2.62) | 1.70 (1.04-2.51) | 0.770 | 1.38 (0.96-1.90) | 1.83 (1.41-2.28) | 1.15 (0.00-1.45) | 0.017 |

**^*^** % represents as a percentage of total lymphocytes. A log_10_ transformation was conducted for IgM titer.

In this analysis, Wilcoxon rank sum test was used. Age groups were divided according to the median age.

**Table S9. Comparison of cellular immunity and humoral immunitybetween sex stratified by age.**

| **Parameters** | **Total**  **(N=86)** | **≤60 years old** | | | **Total**  **(N=108)** | **>60 years old** | | |
| --- | --- | --- | --- | --- | --- | --- | --- | --- |
|  |  | **Female**  **(n=52)** | **Male**  **(n=34)** | ***P* value** |  | **Female**  **(n=61)** | **Male**  **(n=47)** | ***P* value** |
| **B cells (%)** | 10.59 (7.92-15.18) | 10.18 (7.11-11.86) | 11.07 (8.23-15.61) | 0.373 | 9.27 (6.30-12.34) | 10.01 (7.41-12.63) | 7.85 (4.07-10.68) | 0.044 |
| **NK cells (%)** | 12.74 (9.04-19.71) | 11.23 (8.41-20.97) | 13.39 (11.23-17.76) | 0.644 | 21.15 (15.95-27.87) | 18.89 (13.25-24.10) | 22.81 (17.10-38.94) | 0.058 |
| **CD4 cells (%)** | 67.70 (62.97-72.92) | 67.70 (63.00-73.10) | 68.28 (63.36-72.66) | 0.817 | 60.65 (50.72-68.57) | 63.33 (54.56-69.24) | 55.60 (42.20-68.55) | 0.099 |
| **IgM titer** | 1.75 (1.15-2.47) | 1.60 (1.00-2.62) | 1.83 (1.41-2.28) | 0.806 | 1.30 (0.71-2.20) | 1.70 (1.04-2.51) | 1.15 (0.00-1.45) | 0.063 |

**^*^** % represents as a percentage of total lymphocytes. A log_10_ transformation was conducted for IgM titer.

In this analysis, Wilcoxon rank sum test was used. Age groups were divided according to the median age.

**Table S10. Comparison of cytokines and chemokinesbetween age groups stratified by sex.**

| **Parameters** | **Total**  **(N=25)** | **Female** | | | **Total**  **(N=23)** | **Male** | | |
| --- | --- | --- | --- | --- | --- | --- | --- | --- |
|  |  | **≤70 years old**  **(n=15)** | **>70 years old**  **(n=10)** | ***P* value** |  | **≤70 years old**  **(n=11)** | **>70 years old**  **(n=12)** | ***P* value** |
| **IL-1β** | 1.49 (1.44-1.54) | 1.49 (1.44-1.53) | 1.51 (1.45-1.55) | 0.596 | 1.49 (1.44-1.56) | 1.49 (1.42-1.57) | 1.48 (1.44-1.51) | 0.926 |
| **IL-6** | 1.53 (1.34-1.77) | 1.44 (1.29-1.72) | 1.55 (1.49-1.77) | 0.305 | 1.47 (1.33-1.58) | 1.53 (1.47-1.66) | 1.36 (1.19-1.52) | 0.039 |
| **IL-8** | 1.82 (1.69-1.98) | 1.79 (1.64-1.92) | 1.90 (1.80-2.12) | 0.192 | 1.63 (1.57-1.80) | 1.69 (1.63-1.83) | 1.58 (1.56-1.66) | 0.139 |
| **IL-10** | 2.38 (2.20-2.56) | 2.38 (2.09-2.58) | 2.39 (2.25-2.52) | 0.637 | 2.11 (1.98-2.43) | 2.41 (2.28-2.61) | 2.04 (1.94-2.10) | 0.008 |
| **IL-12 p70** | 2.58 (2.46-2.69) | 2.61 (2.55-2.68) | 2.54 (2.43-2.71) | 0.637 | 2.54 (2.46-2.66) | 2.58 (2.50-2.73) | 2.50 (2.45-2.61) | 0.242 |
| **IL-18** | 2.71 (2.65-2.92) | 2.69 (2.64-2.94) | 2.74 (2.68-2.91) | 0.760 | 2.86 (2.75-3.03) | 3.01 (2.82-3.05) | 2.83 (2.66-2.90) | 0.148 |
| **TNF-a** | 1.57 (1.47-1.67) | 1.55 (1.47-1.66) | 1.57 (1.49-1.65) | 0.934 | 1.57 (1.45-1.67) | 1.63 (1.53-1.71) | 1.49 (1.43-1.59) | 0.020 |
| **IFN-r** | 2.36 (2.31-2.59) | 2.34 (2.31-2.61) | 2.39 (2.34-2.55) | 0.802 | 2.45 (2.34-2.55) | 2.55 (2.42-2.60) | 2.36 (2.21-2.52) | 0.074 |
| **CCL2** | 2.93 (2.84-3.14) | 2.91 (2.75-3.13) | 3.07 (2.88-3.14) | 0.375 | 2.99 (2.69-3.15) | 3.07 (2.93-3.24) | 2.76 (2.57-3.08) | 0.045 |
| **CCL5** | 3.90 (3.69-4.14) | 3.85 (3.55-4.14) | 3.92 (3.85-4.02) | 0.620 | 3.97 (3.48-4.06) | 3.62 (3.34-4.00) | 4.05 (4.01-4.08) | 0.439 |
| **CXCL10** | 3.58 (3.32-3.78) | 3.44 (3.26-3.71) | 3.72 (3.52-3.81) | 0.076 | 3.47 (3.30-3.68) | 3.70 (3.50-3.84) | 3.38 (3.29-3.47) | 0.016 |
| **VEGF-A** | 2.03 (1.80-2.09) | 1.99 (1.76-2.09) | 2.03 (1.98-2.11) | 0.571 | 2.01 (1.70-2.12) | 1.70 (1.70-2.01) | 2.12 (2.11-2.14) | 0.241 |
| **P-Selectin** | 4.46 (4.28-4.63) | 4.52 (4.19-4.64) | 4.40 (4.30-4.49) | 0.572 | 4.62 (4.38-4.73) | 4.49 (4.27-4.62) | 4.88 (4.84-4.92) | 0.053 |
| **MMP-9** | 4.66 (4.43-4.89) | 4.57 (4.33-4.83) | 5.09 (4.80-5.36) | 0.090 | 4.87 (4.46-4.94) | 4.51 (4.42-4.90) | 4.93 (4.90-4.96) | 0.439 |
| **Angiopoietin-1** | 4.22 (4.13-4.41) | 4.17 (3.93-4.41) | 4.30 (4.21-4.38) | 0.480 | 4.24 (3.61-4.48) | 3.65 (3.58-4.24) | 4.50 (4.46-4.54) | 0.121 |
| **MRP8** | 3.11 (2.91-3.31) | 3.18 (2.99-3.34) | 3.07 (2.84-3.25) | 0.267 | 3.03 (2.84-3.18) | 3.19 (3.07-3.35) | 2.87 (2.82-3.02) | 0.014 |
| **Angiopoietin-2** | 3.38 (3.24-3.52) | 3.35 (3.21-3.47) | 3.45 (3.33-3.66) | 0.166 | 3.29 (3.16-3.53) | 3.22 (3.06-3.52) | 3.31 (3.18-3.55) | 0.372 |
| **E-Selectin** | 4.27 (4.20-4.42) | 4.31 (4.24-4.44) | 4.24 (4.20-4.36) | 0.318 | 4.37 (4.25-4.52) | 4.38 (4.25-4.53) | 4.37 (4.28-4.47) | 0.829 |
| **GM-CSF** | 2.66 (2.32-2.80) | 2.66 (2.29-2.79) | 2.65 (2.46-2.81) | 0.618 | 2.46 (2.23-2.72) | 2.74 (2.58-2.85) | 2.37 (2.19-2.44) | 0.003 |
| **M-CSF** | 3.94 (3.65-4.50) | 4.15 (3.70-4.42) | 3.90 (3.65-4.45) | 1.000 | 3.70 (3.54-4.45) | 4.38 (3.73-4.55) | 3.57 (3.49-3.72) | 0.023 |
| **G-CSF** | 2.49 (2.17-3.12) | 2.39 (2.14-2.51) | 2.93 (2.53-3.73) | 0.017 | 2.31 (2.23-2.53) | 2.37 (2.23-2.56) | 2.30 (2.22-2.46) | 0.805 |
| **VCAM-1** | 7.18 (6.94-7.33) | 7.18 (6.93-7.28) | 7.14 (6.95-7.34) | 0.718 | 7.17 (6.86-7.35) | 7.31 (6.95-7.36) | 7.10 (6.86-7.26) | 0.538 |
| **ICAM-1** | 5.89 (5.69-6.03) | 5.92 (5.59-6.10) | 5.85 (5.73-6.02) | 0.505 | 5.82 (5.72-5.93) | 5.84 (5.79-5.94) | 5.76 (5.68-5.90) | 0.268 |
| **CD40 Ligand** | 3.59 (3.47-3.71) | 3.64 (3.47-3.72) | 3.56 (3.47-3.65) | 0.331 | 3.58 (3.53-3.72) | 3.60 (3.55-3.66) | 3.58 (3.50-3.76) | 0.853 |

A log_10_ transformation was conducted for all variables as a result of the variability across test patches. In this analysis, Wilcoxon rank sum test was used. Agegroups were divided according to the median age. The unit of 24 cytokines and chemokines in the study is picograms/milliliter (pg/mL).

**Table S11. Comparison of cytokines and chemokinesbetween sex stratified by age.**

| **Parameters** | **Total**  **(N=26)** | **≤70 years old** | | | **Total**  **(N=22)** | **>70 years old** | | |
| --- | --- | --- | --- | --- | --- | --- | --- | --- |
|  |  | **Female**  **(n=15)** | **Male**  **(n=11)** | ***P* value** |  | **Female**  **(n=10)** | **Male**  **(n=12)** | ***P* value** |
| **IL-1β** | 1.49 (1.44-1.55) | 1.49 (1.44-1.53) | 1.49 (1.42-1.57) | 0.835 | 1.49 (1.44-1.55) | 1.51 (1.45-1.55) | 1.48 (1.44-1.51) | 0.714 |
| **IL-6** | 1.50 (1.34-1.69) | 1.44 (1.29-1.72) | 1.53 (1.47-1.66) | 0.337 | 1.49 (1.32-1.63) | 1.55 (1.49-1.77) | 1.36 (1.19-1.52) | 0.029 |
| **IL-8** | 1.74 (1.63-1.91) | 1.79 (1.64-1.92) | 1.69 (1.63-1.83) | 0.406 | 1.70 (1.57-1.97) | 1.90 (1.80-2.12) | 1.58 (1.56-1.66) | 0.056 |
| **IL-10** | 2.39 (2.16-2.61) | 2.38 (2.09-2.58) | 2.41 (2.28-2.61) | 0.795 | 2.11 (1.99-2.40) | 2.39 (2.25-2.52) | 2.04 (1.94-2.10) | 0.003 |
| **IL-12 p70** | 2.59 (2.54-2.69) | 2.61 (2.55-2.68) | 2.58 (2.50-2.73) | 0.938 | 2.50 (2.43-2.67) | 2.54 (2.43-2.71) | 2.50 (2.45-2.61) | 0.766 |
| **IL-18** | 2.86 (2.66-3.02) | 2.69 (2.64-2.94) | 3.01 (2.82-3.05) | 0.052 | 2.80 (2.68-2.91) | 2.74 (2.68-2.91) | 2.83 (2.66-2.90) | 0.792 |
| **TNF-a** | 1.57 (1.49-1.71) | 1.55 (1.47-1.66) | 1.63 (1.53-1.71) | 0.377 | 1.55 (1.45-1.60) | 1.57 (1.49-1.65) | 1.49 (1.43-1.59) | 0.274 |
| **IFN-r** | 2.42 (2.34-2.61) | 2.34 (2.31-2.61) | 2.55 (2.42-2.60) | 0.161 | 2.36 (2.27-2.54) | 2.39 (2.34-2.55) | 2.36 (2.21-2.52) | 0.448 |
| **CCL2** | 2.96 (2.78-3.19) | 2.91 (2.75-3.13) | 3.07 (2.93-3.24) | 0.287 | 2.99 (2.68-3.14) | 3.07 (2.88-3.14) | 2.76 (2.57-3.08) | 0.086 |
| **CCL5** | 3.80 (3.50-4.12) | 3.85 (3.55-4.14) | 3.62 (3.34-4.00) | 0.391 | 3.96 (3.91-4.08) | 3.92 (3.85-4.02) | 4.05 (4.01-4.08) | 0.355 |
| **CXCL10** | 3.64 (3.30-3.78) | 3.44 (3.26-3.71) | 3.70 (3.50-3.84) | 0.146 | 3.47 (3.31-3.65) | 3.72 (3.52-3.81) | 3.38 (3.29-3.47) | 0.006 |
| **VEGF-A** | 1.91 (1.70-2.09) | 1.99 (1.76-2.09) | 1.70 (1.70-2.01) | 0.540 | 2.07 (2.02-2.14) | 2.03 (1.98-2.11) | 2.12 (2.11-2.14) | 0.355 |
| **P-Selectin** | 4.49 (4.20-4.64) | 4.52 (4.19-4.64) | 4.49 (4.27-4.62) | 1.000 | 4.49 (4.36-4.72) | 4.40 (4.30-4.49) | 4.88 (4.84-4.92) | 0.064 |
| **MMP-9** | 4.56 (4.35-4.88) | 4.57 (4.33-4.83) | 4.51 (4.42-4.90) | 0.713 | 4.93 (4.86-5.23) | 5.09 (4.80-5.36) | 4.93 (4.90-4.96) | 1.000 |
| **Angiopoietin-1** | 4.13 (3.72-4.40) | 4.17 (3.93-4.41) | 3.65 (3.58-4.24) | 0.391 | 4.39 (4.27-4.42) | 4.30 (4.21-4.38) | 4.50 (4.46-4.54) | 0.165 |
| **MRP8** | 3.18 (3.04-3.35) | 3.18 (2.99-3.34) | 3.19 (3.07-3.35) | 0.856 | 2.99 (2.79-3.11) | 3.07 (2.84-3.25) | 2.87 (2.82-3.02) | 0.176 |
| **Angiopoietin-2** | 3.33 (3.11-3.51) | 3.35 (3.21-3.47) | 3.22 (3.06-3.52) | 0.716 | 3.37 (3.22-3.64) | 3.45 (3.33-3.66) | 3.31 (3.18-3.55) | 0.198 |
| **E-Selectin** | 4.37 (4.25-4.46) | 4.31 (4.24-4.44) | 4.38 (4.25-4.53) | 0.392 | 4.32 (4.20-4.40) | 4.24 (4.20-4.36) | 4.37 (4.28-4.47) | 0.146 |
| **GM-CSF** | 2.71 (2.35-2.85) | 2.66 (2.29-2.79) | 2.74 (2.58-2.85) | 0.452 | 2.43 (2.25-2.59) | 2.65 (2.46-2.81) | 2.37 (2.19-2.44) | 0.008 |
| **M-CSF** | 4.24 (3.68-4.52) | 4.15 (3.70-4.42) | 4.38 (3.73-4.55) | 0.378 | 3.67 (3.54-3.93) | 3.90 (3.65-4.45) | 3.57 (3.49-3.72) | 0.052 |
| **G-CSF** | 2.38 (2.16-2.54) | 2.39 (2.14-2.51) | 2.37 (2.23-2.56) | 0.815 | 2.47 (2.27-3.02) | 2.93 (2.53-3.73) | 2.30 (2.22-2.46) | 0.025 |
| **VCAM-1** | 7.22 (6.91-7.35) | 7.18 (6.93-7.28) | 7.31 (6.95-7.36) | 0.517 | 7.10 (6.90-7.34) | 7.14 (6.95-7.34) | 7.10 (6.86-7.26) | 0.598 |
| **ICAM-1** | 5.90 (5.74-6.03) | 5.92 (5.59-6.10) | 5.84 (5.79-5.94) | 0.640 | 5.85 (5.69-5.98) | 5.85 (5.73-6.02) | 5.76 (5.68-5.90) | 0.597 |
| **CD40 Ligand** | 3.62 (3.53-3.71) | 3.64 (3.47-3.72) | 3.60 (3.55-3.66) | 0.959 | 3.57 (3.47-3.68) | 3.56 (3.47-3.65) | 3.58 (3.50-3.76) | 0.373 |

A log_10_ transformation was conducted for all variables as a result of the variability across test patches. In this analysis, Wilcoxon rank sum test was used. Agegroups were divided according to the median age. The unit of 24 cytokines and chemokines in the study is picograms/milliliter (pg/mL).
