# Supplementary figures and images for: Sex Differences in Case Fatality Rate of Patients With Severe Fever With Thrombocytopenia Syndrome
Source: Front Microbiol. 2021 Oct 14;12:738808. doi: 10.3389/fmicb.2021.738808 (PMC8552034; doi:10.3389/fmicb.2021.738808)

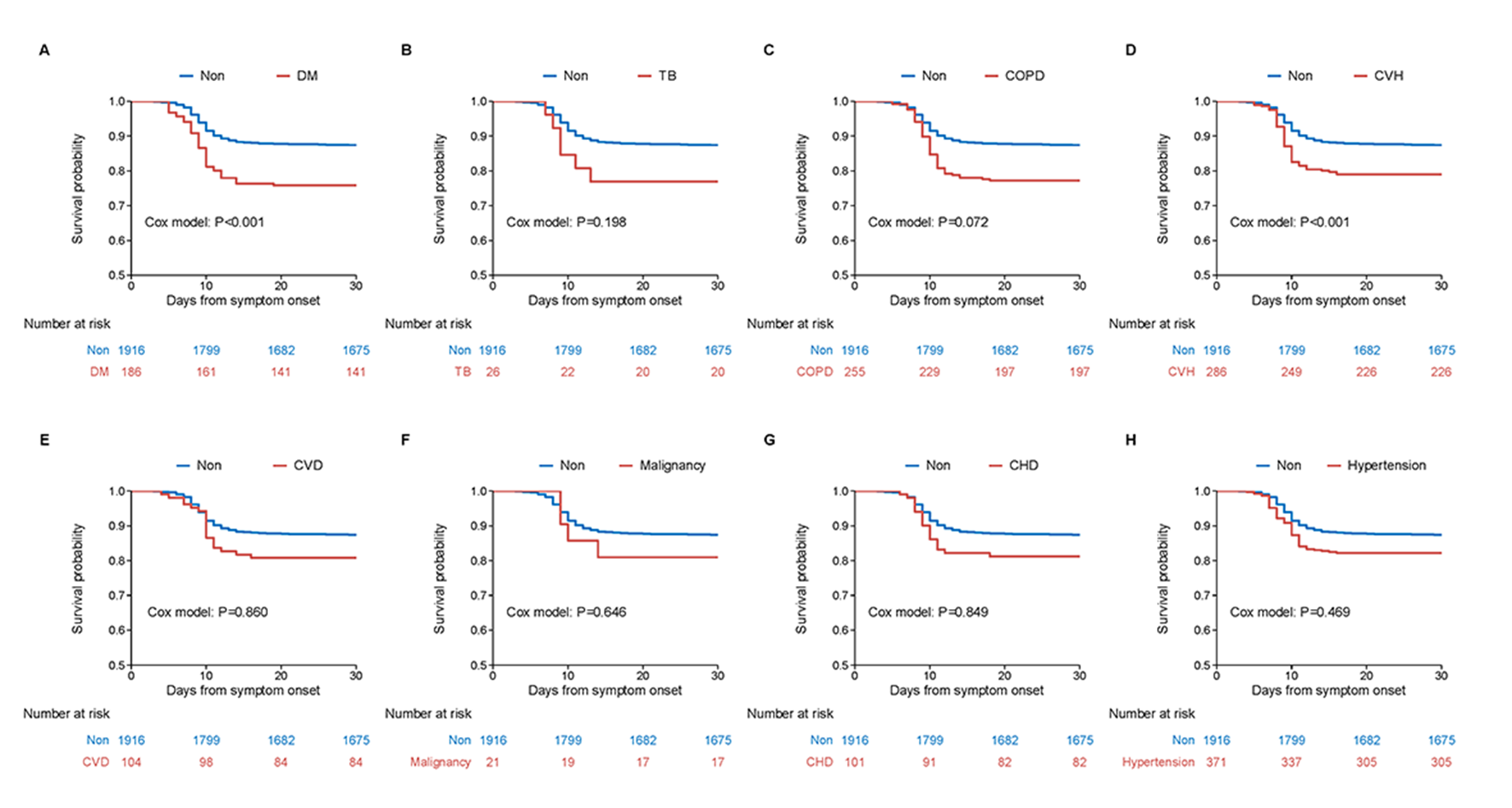

Supplement: Supplementary Figure 1 — Kaplan–Meier survival curves for specific comorbidity. P value was calculated using multiple Cox regression model, adjusting for age, sex, and delay from symptom onset to hospital admission. The numbers of patients without comorbidity and with specific comorbidity across days from symptom onset were listed on the below. The blue lines and numbers indicate non-comorbidity. The red lines and numbers indicate specific comorbidity. [file Image_1.TIFF]

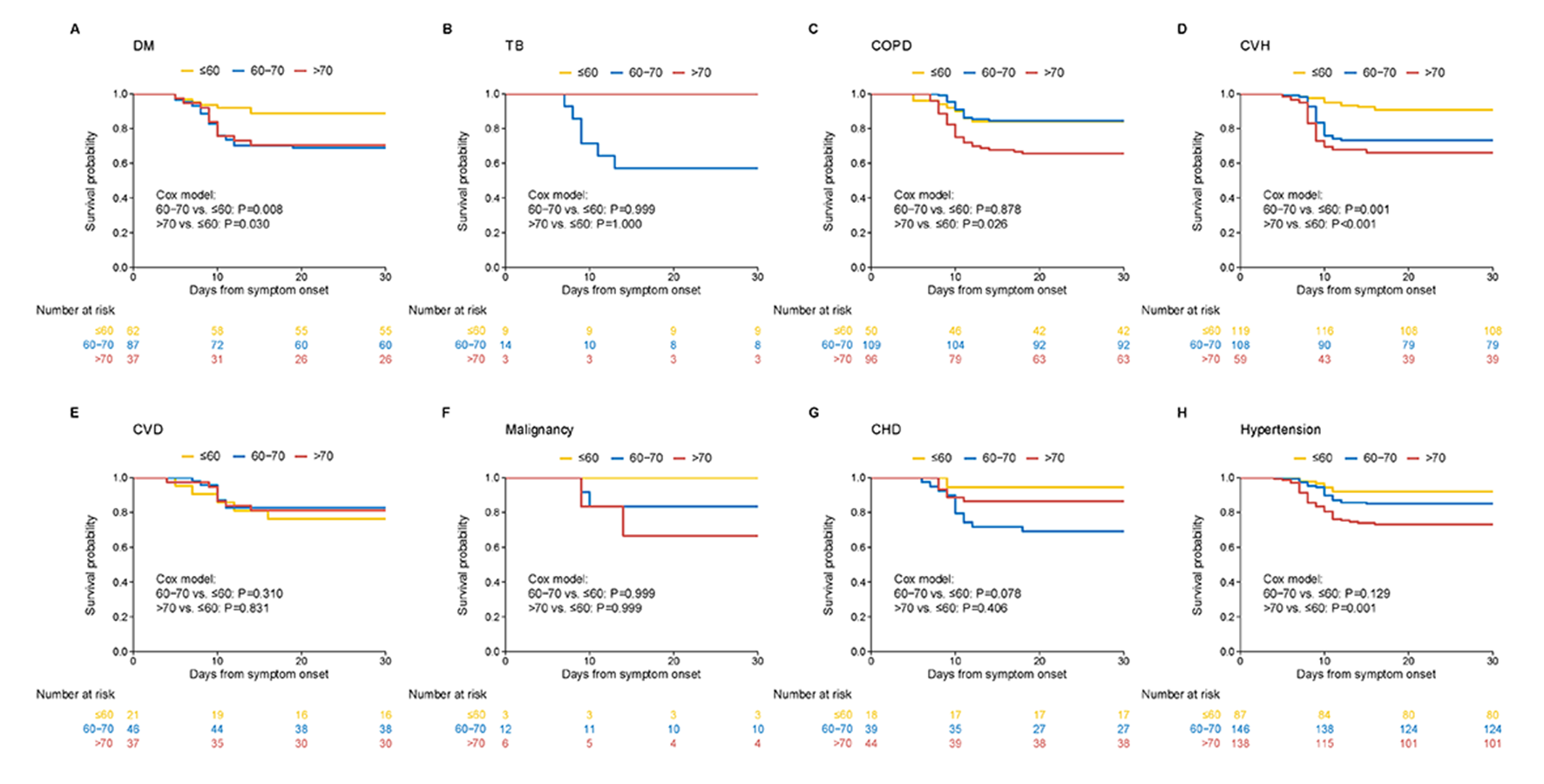

Supplement: Supplementary Figure 2 — Kaplan–Meier survival curves based on age stratification for specific comorbidity. P value was calculated using multiple Cox regression model, adjusting for sex and delay from symptom onset to hospital admission. The numbers of patients age ≤60 years, 60–70 years, and >70 years across days from symptom onset were listed on the below. The blue lines and numbers indicate age ≤60 years, the yellow lines and numbers indicate 60–70 years, and the red lines and numbers indicate >70 years. [file Image_2.TIFF]

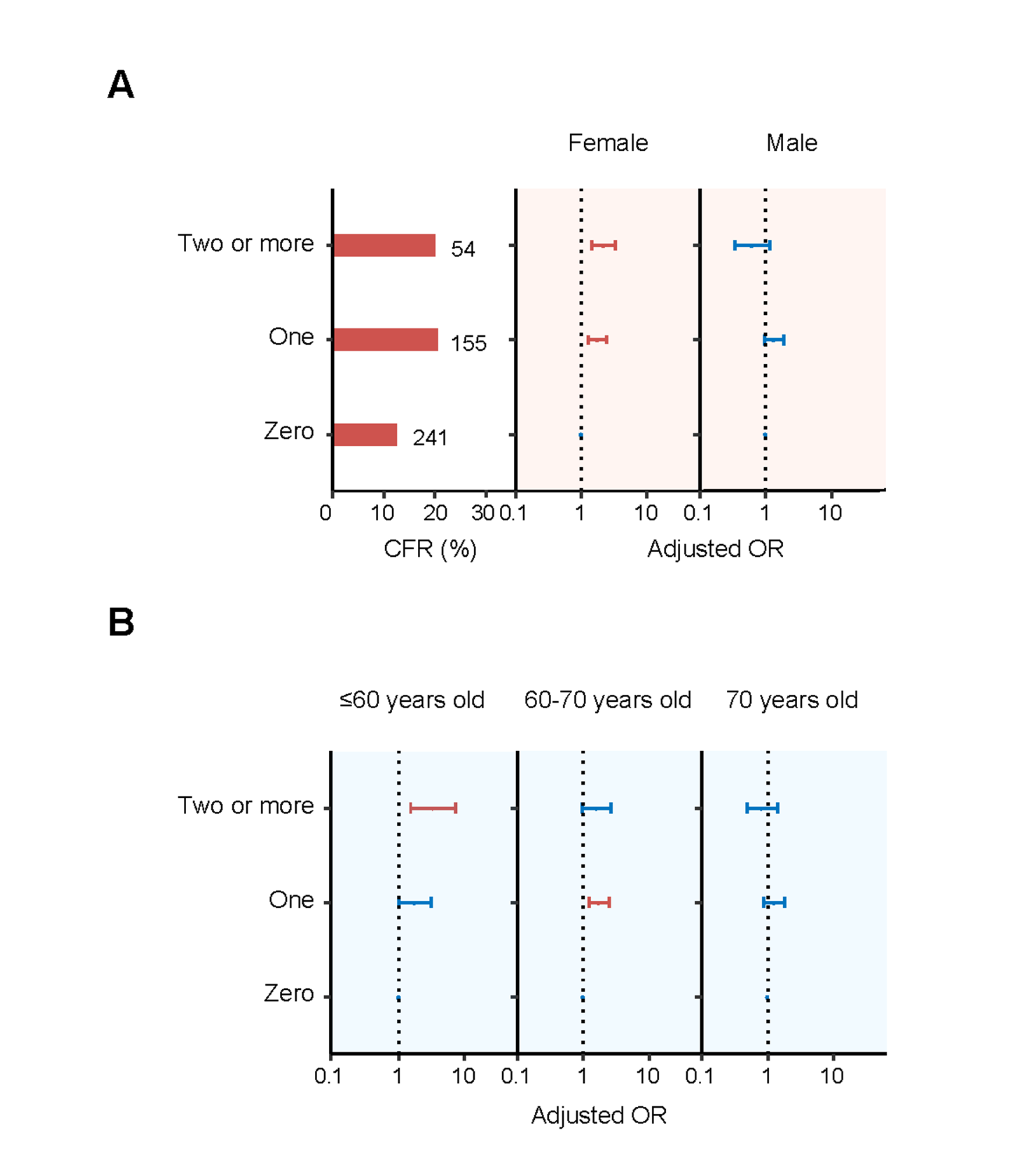

Supplement: Supplementary Figure 3 — Presence of multiple coexisting comorbidities and risk for fatal outcome of SFTS patients stratified by sex (A) or age (B). The numbers of deaths and CFRs of the SFTS patients with multiple coexisting comorbidities were shown to the left of column in panel (A). Multivariate logistic regression model was performed for association between comorbidity and fatality by adjusting age, sex, and delay from symptom onset to hospital admission. Adjusted ORs (aORs) and 95% CIs were presented for multiple coexisting comorbidities. The dots are the aORs and the error bars are the 95% CIs. The red color represents P < 0.05 and the blue color represents P ≥ 0.05. The dotted line indicates an OR of 1. [file Image_3.TIFF]

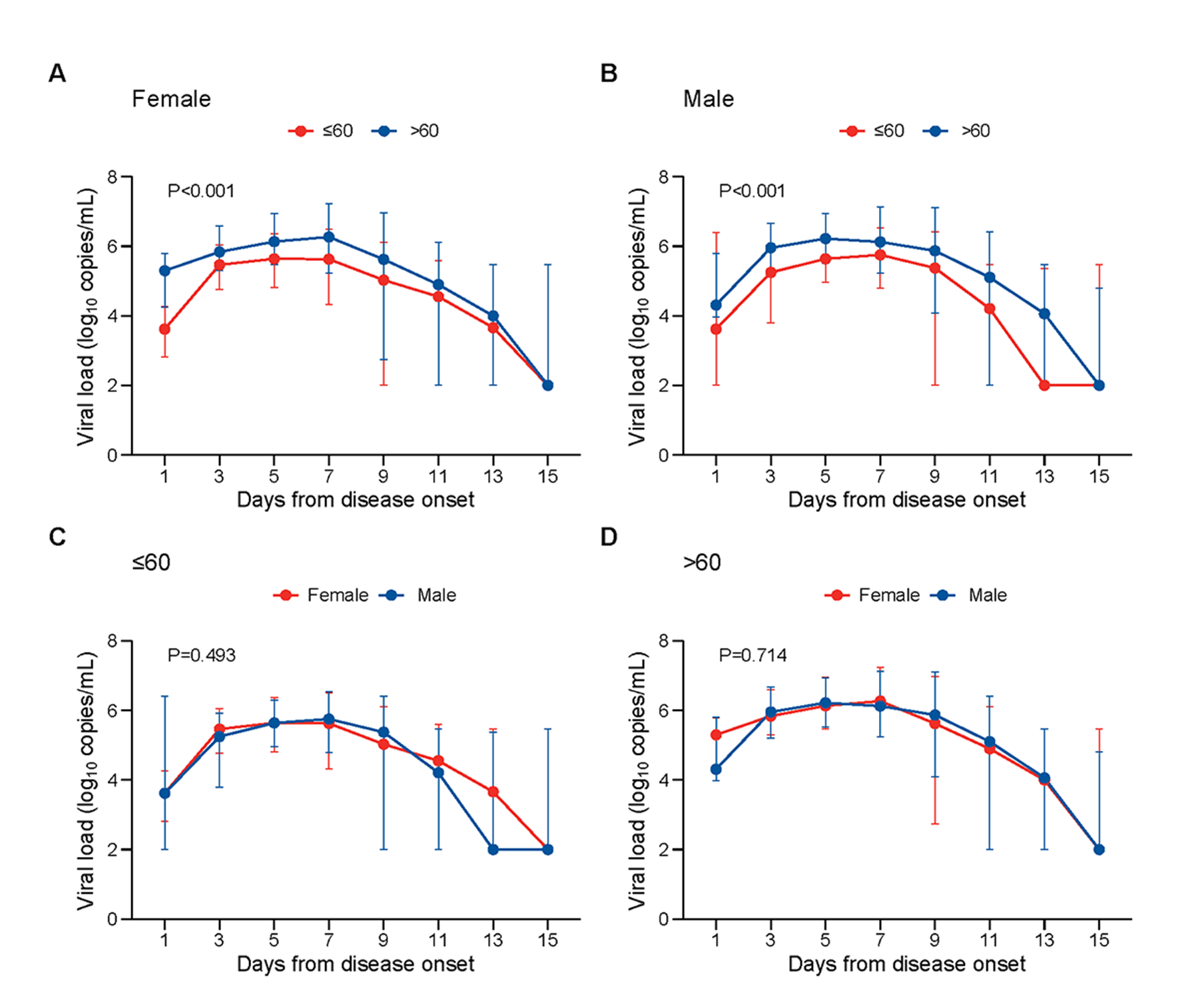

Supplement: Supplementary Figure 4 — Kinetics of viral load stratified by sex and age in SFTS patients. P values were calculated by generalized estimating equation model. The adjusted variables were delay from symptom onset to hospital admission and with any one of comorbidity. Viral load was measured as log10 copies/mL. Age groups were classified by median value. The dots and the error bars denote medians and IQRs. P < 0.05 indicates statistically significant. [file Image_4.TIFF]
